# Rapid physiological and transcriptomic changes associated with oxygen delivery in larval anemonefish suggest a role in adaptation to life on hypoxic coral reefs

**DOI:** 10.1371/journal.pbio.3002102

**Published:** 2023-05-11

**Authors:** Adam T. Downie, Sjannie Lefevre, Björn Illing, Jessica Harris, Michael D. Jarrold, Mark I. McCormick, Göran E. Nilsson, Jodie L. Rummer

**Affiliations:** 1 Australian Research Council Centre of Excellence for Coral Reef Studies, James Cook University, Townsville, Australia; 2 School of Biological Sciences, University of Queensland, St. Lucia, Australia; 3 Section for Physiology and Cell Biology, Department of Biosciences, University of Oslo, Oslo, Norway; 4 Thünen Institute of Fisheries Ecology, Bremerhaven, Germany; 5 College of Science and Engineering, James Cook University, Townsville, Australia; 6 Coastal Marine Field Station, School of Science, University of Waikato, Tauranga, New Zealand; University of Washington Seattle Campus: University of Washington, UNITED STATES

## Abstract

Connectivity of coral reef fish populations relies on successful dispersal of a pelagic larval phase. Pelagic larvae must exhibit high swimming abilities to overcome ocean and reef currents, but once settling onto the reef, larvae transition to endure habitats that become hypoxic at night. Therefore, coral reef fish larvae must rapidly and dramatically shift their physiology over a short period of time. Taking an integrative, physiological approach, using swimming respirometry, and examining hypoxia tolerance and transcriptomics, we show that larvae of cinnamon anemonefish (*Amphiprion melanopus*) rapidly transition between “physiological extremes” at the end of their larval phase. Daily measurements of swimming larval anemonefish over their entire early development show that they initially have very high mass-specific oxygen uptake rates. However, oxygen uptake rates decrease midway through the larval phase. This occurs in conjunction with a switch in haemoglobin gene expression and increased expression of myoglobin, cytoglobin, and neuroglobin, which may all contribute to the observed increase in hypoxia tolerance. Our findings indicate that critical ontogenetic changes in the gene expression of oxygen-binding proteins may underpin the physiological mechanisms needed for successful larval recruitment to reefs.

## Abstract 2

Connectivity of coral reef fish populations relies on successful dispersal of a pelagic larval phase. This study shows that anemonefish larvae exhibit physiological and transcriptomic changes associated with swimming, oxygen uptake and hypoxia tolerance; these changes potentially support dispersal and connectivity among coral reefs.

## Introduction

**Fig 1:**
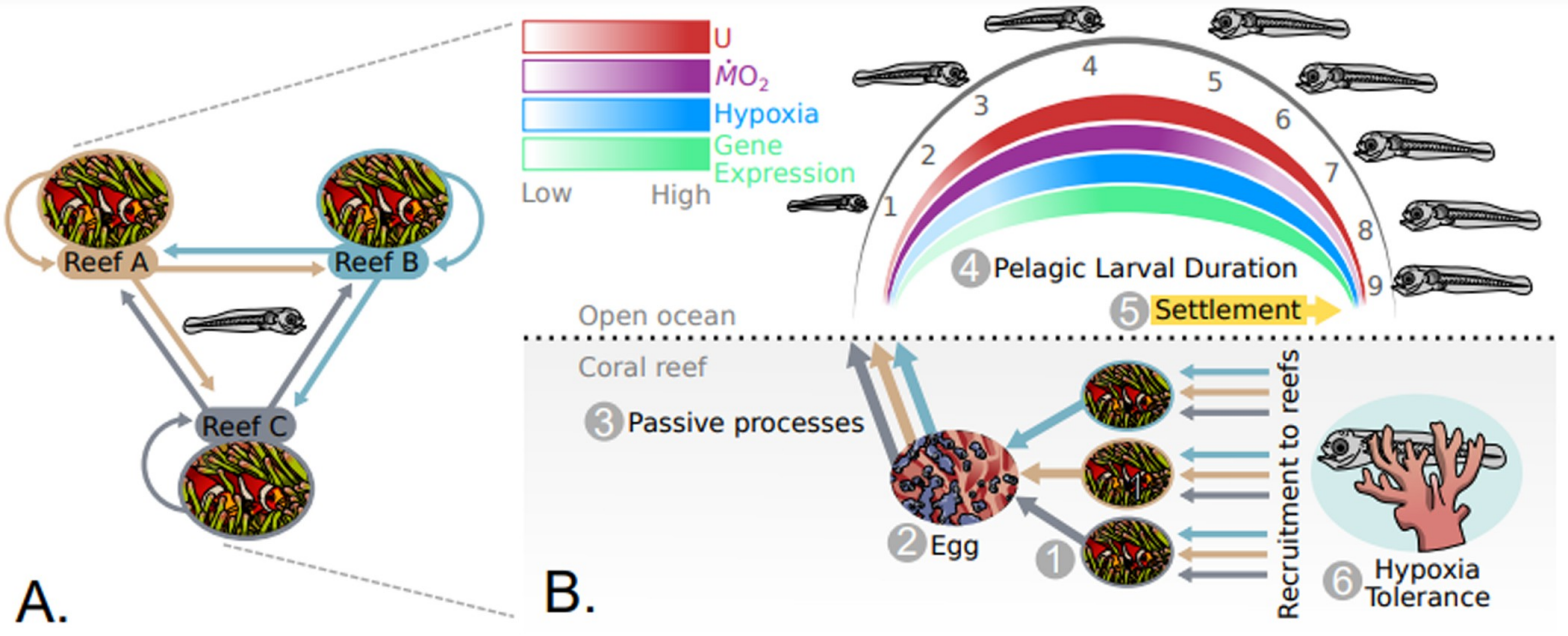
Conceptual framework of connectivity and life history of reef fishes, along with *a priori* predictions for how the physiology of reef fishes changes during early life history. (**A**) Reef fish populations exist in patches on reefs isolated by swift currents and depth (grey, pink, and blue reefs indicate separate, isolated reef fish populations). Connectivity between populations occurs via a dispersive pelagic larval phase, capable of fast swimming speeds and well-developed sensory systems and behaviours. Straight arrows indicate movement of larvae between reefs, and curved arrows indicate self-recruitment back to natal reefs. (**B**) The general life history of reef fishes. Adult reef fishes living on benthic reef habitats (1), lay their eggs on the reef (2), which are carried off the reef by passive processes like currents and wave energy (3). The eggs hatch, and the larvae undergo a pelagic larval phase (9 days for focal cinnamon anemonefish (*Amphiprion melanopus)*, as indicated by numbers under each larva) in the open ocean (4). The reef fish must quickly develop and grow before swimming back to the reef during this phase, and we predict that mass-specific oxygen uptake rates (*Ṁ*O_2_, a proxy for metabolic rates) are very high for most of the larval duration (indicated by purple gradient). Swimming performance measured as the critical swimming speed (U_crit_) is predicted to be poor during the first few dph but increases as fish grow and develop (indicated by red gradient). Hypoxia tolerance is predicted to increase over larval development during their pelagic phase, which is thought to enable low-oxygen level challenges on the reef at night (indicated by blue gradient). Reef fish larvae settle onto the reef (5) but are already remarkably hypoxia tolerant (low oxygen due to nocturnal coral respiration) and are predicted to lower their oxygen uptake rates to become suitably hypoxia tolerant (indicated by purple gradient). Changes in oxygen uptake rates, swimming performance, and hypoxia tolerance are predicted to be related to gene expression patterns of proteins responsible for oxygen transport and delivery to tissues (e.g., Hb, Mb, Ngb, and Cytgb; indicated by green gradient). The combination of fast swimming speeds during pelagic phases and predicted changing metabolic demands to tolerate hypoxia in new reef habitats (new or natal reef; refer to Fig 1A) may be selected for to support connectivity and recruitment of larvae to adult populations (6). Cytgb, cytoglobin; dph, days post hatch; Hb, haemoglobin; Mb, myoglobin; Ngb, neuroglobin.

Tropical coral reef fishes are among the most biodiverse (6,000 to 8,000 species) and evolutionary derived group of fishes [1] and play critical roles in maintaining coral reef health and resilience to environmental change [2]. However, despite the diversity of colours, forms, and functions, the life history of most of these fishes are largely similar. Adult reef fishes are specialised for reef life and are generally site-attached to a small patch of reef and therefore do not move large distances between reefs [3,4]. With very few exceptions, connectivity between reefs is achieved by a dispersive pelagic larval phase, whereby larvae develop in the open ocean over a short period of time (9 to 50 days on average, depending on species, compared with temperate species that develop over several months) and swim back to natal or novel reefs [3,4] (Fig 1). To be able to swim against ocean currents, high swimming performance has presumably been selected for over evolutionary history. Indeed, these larvae are capable of incredible swimming speeds under controlled laboratory conditions and *in situ* [5–8], reaching 10 to 50 body lengths (BLs) s^−1^ (species-specific) compared with temperate species larvae that swim < 5 BL s^−1^ at similar stages of development. The high swimming speeds of coral reef fish larvae are supported by the highest maximal mass-specific oxygen uptake rates (*Ṁ*O_2_) of any teleost fish species (e.g., *Chromis atripectoralis Ṁ*O_2_ = 5,250 mg O_2_ kg^−1^ h^−1^; [9]). This performance, in conjunction with advanced sensory systems (e.g., visual, auditory, and olfactory) of young fishes, makes them well-adapted to detect and swim towards reefs [4]. Despite these advantages, reef life poses a unique challenge for newly settled reef fishes.

Coastal habitats generally experience large daily fluctuations in several environmental conditions, including temperature, salinity, and oxygen levels [10]. Coral reefs become hypoxic at night due to animal and plant respiration [9,11]. Upon settlement onto a reef, juvenile reef fishes across several species are capable of tolerating oxygen levels as low as 10% to 30% of air saturation [12]. Therefore, hypoxia tolerance among fishes living on coral reefs can be considered an environmental adaptation [11], where specific physiological traits enable them to survive under nocturnal low oxygen levels (Fig 1). Generally, however, hypoxia-tolerant fishes (e.g., carp) are relatively poor swimmers and have low oxygen uptake rates compared to more athletic fishes (e.g., salmon and tuna) that are sensitive to low oxygen [13]. There are several well-studied selection-driven physiological modifications that lead to hypoxia tolerance among fishes, including increased blood oxygen binding affinity, decreased respiration rates, lower tissue oxygen demands, and other adaptations related to oxygen transport and storage [10]. There is a well-established trade-off between having high maximal rates of oxygen uptake and being hypoxia tolerant, and there are no examples of animals that simultaneously can achieve both [9,11]. A major reason for this is that the former demands low-affinity haemoglobin (Hb) that readily releases oxygen to the tissues, while hypoxia tolerance requires high-affinity Hb to be able to take up oxygen when environmental levels are low [9].

Unlike mammals, many fishes possess multiple Hbs and can change the overall oxygen binding affinity of the blood (i.e., via Hb protein subunit substitution) in response to environmental and developmental cues [14–16]. While Hb is the primary protein responsible for oxygen transport from the environment to the tissues, there are also other globins capable of binding oxygen. Myoglobin (Mb) has a high oxygen binding affinity and is generally found in tissues with high oxidative properties and is considered the primary protein used for oxygen storage in skeletal muscles and heart tissue [17]. Neuroglobin (Ngb) found in brain tissue in some fishes has been described as a “neural myoglobin,” and its proposed function is enhanced oxygen delivery to neurons during low oxygen scenarios [18]. Cytoglobin (Cytgb) has been predicted to act as an oxygen store for connective tissues in mammals as well as a proposed antioxidant for the liver, retina, and kidney [19]. Taken together, a proposed mechanism that may support hypoxia tolerance among coral reef fishes, and which would also be the simplest explanation, is the switch in Hb from low to high affinity [9], as this would allow the juvenile and adult fish to maintain oxygen uptake in hypoxia. This may be accompanied by increased expression of Mb, Ngb, and Cytgb serving as oxygen stores so that oxygen is not depleted from these tissues under nocturnal hypoxic events on the reef. These possible changes in globin gene expression have yet to be characterised for any coral reef fish species but may compliment the challenge of being both an aerobic “athlete” in the pelagic and hypoxia tolerant upon reaching a reef.

Given that the physiological traits necessary for high swimming performance would primarily be aerobically driven, such traits would form the basis of their ability to disperse among reefs, which, in turn, would contribute to connectivity. Therefore, the aerobic demands of pelagic larvae would predictably be very high in order to support fast growth, swimming capabilities, and development, within their very narrow ontogenetic window. Measuring such changes would require creating a profile for oxygen uptake rates during activity, since continuous swimming is critical for pelagic larvae to find a reef. This profile would need to encompass the entire larval phase of a reef fish to accurately identify periods of change. Such profiles do not currently exist for any other fish species, but would clearly show how oxygen demands change over early ontogeny, and may correlate with other physiological changes as development proceeds to cope with hypoxia on the reef.

Consequently, the aim of the current study was to measure representative physiological traits that characterise the early life history of a representative coral reef fish, the cinnamon anemonefish (*Amphiprion melanopus*). The traits of interest are those that support high speed swimming and those that prepare the fish for hypoxic reef conditions. The critical knowledge gaps we aim to fill include determining the energy demands during the pelagic phase that support “athletic” swimming performance and highlighting the changes in gene expression for proteins that can enhance oxygen uptake, transport, and storage and that are therefore needed to support hypoxia tolerance upon settlement. To achieve this, we measured mass-specific oxygen uptake rates at increasing swimming speeds every day over the entire larval duration of cinnamon anemonefish. We hypothesised that mass-specific oxygen uptake rates would be high when larvae need a high aerobic capacity to support both high swimming performance and rapid development within a narrow larval growth window (9 days as a pelagic larva for the cinnamon anemonefish; Fig 1). Near the end of their pelagic phase, we hypothesised that the larvae would show a decrease in oxygen uptake rates to accommodate high affinity oxygen uptake in the hypoxic environment they are about to encounter (Fig 1). Observed changes in oxygen uptake were used to design the hypoxia tolerance tests and collection of larvae to measure changes in gene expression. We hypothesised that hypoxia tolerance would improve over larval development and expression of Hb isoforms, and, possibly, expression of other globins (Mb, Cytgb, and Ngb) would show adjustments in preparation for the hypoxic conditions on coral reefs (Fig 1).

## Results

Using a representative reef fish species, our study characterised some of the physiological changes that pelagic reef fish larvae may undergo to enable them to swim back to and settle onto a reef. Specifically, we found that larvae of the cinnamon anemonefish undergo changes in oxygen uptake rates and gene expression patterns of some key proteins proposed to be responsible for oxygen transport, delivery, and storage, prior to settling onto a reef, changes that are presumed to support both high swimming performance and hypoxia tolerance. To our knowledge, these larvae have the highest mass-specific oxygen uptake rates of any teleost in the published literature, which may be associated with their rapid development and high swimming performance. Additionally, decreases in mass-specific oxygen uptake rates midway through the larval phase coincided with progressively increased hypoxia tolerance. The change in hypoxia tolerance was also correlated with switching in the gene expression for Hb isoforms and increasing mRNA expression of Mb, Cytgb, and Ngb genes during the onset of hypoxia tolerance and prior to settlement onto the reef.

### Changes in swimming performance and oxygen uptake rates over early ontogeny

**Fig 2:**
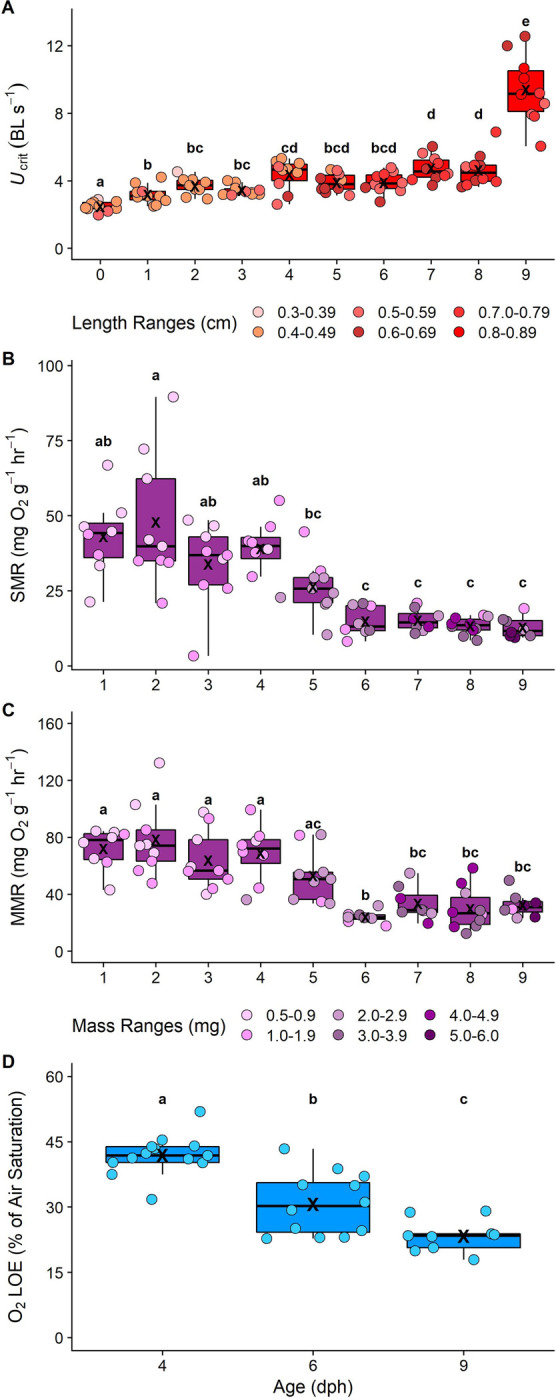
The relationship between (**A**) critical swimming speed (*U*_*crit*_; BL s^−1^), (**B**) SMR (mg O_2_ g^−1^ h^−1^), (**C**) MMR (mg O_2_ g^−1^ h^−1^), and (**D**) hypoxia tolerance (O_2,LOE_; % of air saturation) with age (dph). *U*_crit_, SMR, and MMR were measured over the entire larval phase of the cinnamon anemonefish (*Amphiprion melanopus*), and hypoxia tolerance was measured during the period where oxygen uptake rates decreased (i.e., 4 and 6 dph) and prior to settlement (i.e., 9 dph). *U*_*crit*_, SMR, and MMR were measured using a swimming respirometry protocol (see Methods for details) to achieve simultaneous measures of swimming speed and oxygen uptake rates. Hypoxia tolerance is represented as the % of air saturation that results in LOE; see Methods for details. Symbols represent the data for an individual larva (*n =* 8–10 individuals for *U*_*crit*_, SMR, and MMR; hypoxia tolerance: *n* = 13 larvae for 4 dph, *n* = 12 larvae for 6 dph, and *n* = 9 larvae for 9 dph). Each data point for *U*_*crit*_, SMR, and MMR is colour coded to represent the size range of individual larva (length: cm; or mass: mg). For *U*_*crit*_, SMR, MMR, and hypoxia tolerance, boxplots show median and interquartile ranges; “X” indicates averages at each age (dph), and different lowercase letters represent statistical differences (LMs; α = 0.05). The data underlying this figure can be found in sheets 1, 2, and 3 in S1 Data, and details on statistical output can be found in S1 StatisticalOutput. dph, days post hatch; LM, linear model; LOE, loss of equilibrium; MMR, maximum metabolic rate; SMR, standard metabolic rate.

Respirometry was carried out in combination with a critical swimming speed protocol to determine *U*_crit_, which is a measure of maximum swimming speed that can be maintained primarily aerobically using a forced stepwise increase in water velocity in a swimming chamber until the fish fatigued (see “Experimental protocol” in Methods for more details). Daily changes in oxygen uptake rates and swimming performance were measured over the entire larval phase of the cinnamon anemonefish. This fine-scale profile allowed us to identify age(s) (days post hatch (dph)) when ontogenetic changes in these parameters occurred in relation to timing of settlement on the reef. Swimming performance (Fig 2A) increased significantly each day from 2.48 ± 0.28 BL s^−1^ at hatch to 9.4 ± 1.9 BL s^−1^ at the point of settlement at 9 dph (mean ± SD; *p* < 0.001; see S2 Fig for *U*_*crit*_ in cm s^−1^ versus age).

Mass-specific oxygen uptake rates (*Ṁ*O_2_; mg O_2_ g^−1^ h^−1^) were measured to estimate both standard metabolic rate (SMR, i.e., the oxygen uptake at rest) and maximum metabolic rate (MMR, i.e. the oxygen uptake under maximal aerobic exercise). These data allowed us to calculate both absolute aerobic scope (AAS; the difference between MMR and SMR, representing an organism’s ability to increase their oxygen uptake rates above rest) and factorial aerobic scope (FAS; the ratio of MMR to SMR, representing how many-fold an organism can increase its oxygen uptake rates above rest). MMR was estimated from oxygen uptake rates at the highest swimming speeds before fatigue and SMR as the y-intercept at zero swimming speed (x-axis) when a best-fit line was plotted through the relationship of swimming speed and oxygen uptake (see “Experimental protocol” in Methods for details). We predicted, *a priori*, that decreases in SMR and MMR during the larval phase may indicate critical periods when globin expression patterns progressively change, potentially contributing to increased hypoxia tolerance. Over early ontogeny, we found significant decreases in mass-specific SMR (linear model (LM), *p* < 0.001, F_8,68_ = 13.49, r^2^ = 0.61; Fig 2B) and mass-specific MMR (LM, *p* < 0.001, F_8,68_ = 13.19, r^2^ = 0.6; Fig 2C) at specific ages, since mass and age are confounded (S1 Fig). Following a similar pattern to each other, mass-specific SMR and MMR remained the same for the first 4 dph (LM, *p* > 0.5 for all combinations; Fig 2B and 2C) before decreasing at 4 dph until 6 dph (LM, *p* > 0.2 for all combinations comparing 1 to 4 dph until 6 dph; Fig 2B and 2C). Both mass-specific SMR and MMR remained consistently lower from 6 dph until 9 dph (i.e., the point of settlement) when compared to fish larvae aged 1 to 4 dph (LM, *p* < 0.02 for all combinations; Fig 2B and 2C). We determined that the SMR and MMR of larger individuals were even lower than would be expected from more commonly observed scaling relationships (S3 and S4 Figs).

Neither AAS (mg O_2_ g^−1^ h^−1^) nor FAS changed significantly over the larval period (S5A and S5B Fig). There was a significant effect of age on AAS (LM, *p* = 0.0012) from the model output, but this was likely due to the decrease in AAS at 6 dph. Further, emmeans *post hoc* tests did not reveal any significant changes in AAS over age (see supporting information S1 StatisticalOutput for details).

### Changes in hypoxia tolerance over early ontogeny

We performed hypoxia tolerance experiments at the ages where we found significant decreases in oxygen uptake rates (between 4 dph and 6 dph), and also at the age where larvae settle and would need to be hypoxia tolerant (9 dph). We selected these ages because we predicted, *a priori*, that measured decreases in oxygen uptake rates may indicate periods in the pelagic larval stage when hypoxia tolerance begins to develop prior to settlement. As the larval phase progressed, fish became significantly more hypoxia tolerant (LM, F_2,31_ = 32.53, *p* < 0.001, r^2^ = 0.68; Fig 2D). At 4 dph, larvae tolerated oxygen levels down to 42.0 ± 4.6 (mean ± SD) % of air saturation (corresponding to 2.75 mg O_2_ L^−1^ and PO_2_ = 64.2 mm Hg at 28°C) before loss of equilibrium (LOE) ensued (Fig 2D). At 6 dph, the point where oxygen uptake rates decreased significantly, larvae could tolerate even lower oxygen levels of 30.7 ± 7.1% of air saturation (i.e., 1.99 mg O_2_ L^−1^; PO_2_ = 46.93 mm Hg at 28°C) before LOE ensued (Fig 2D). Upon settlement, larvae tolerated dissolved oxygen levels that were as low as 23.8 ± 3.7% of air saturation (i.e., 1.53 mg O_2_ L^−1^; PO_2_ = 36.38 mm Hg at 28°C) before LOE ensued; this was 44.2% of air saturation lower than their 4 dph counterparts and 23.8% of air saturation lower than their 6 dph counterparts (Fig 2D).

### Changes in gene expression related to oxygen transport over early ontogeny

**Fig 3:**
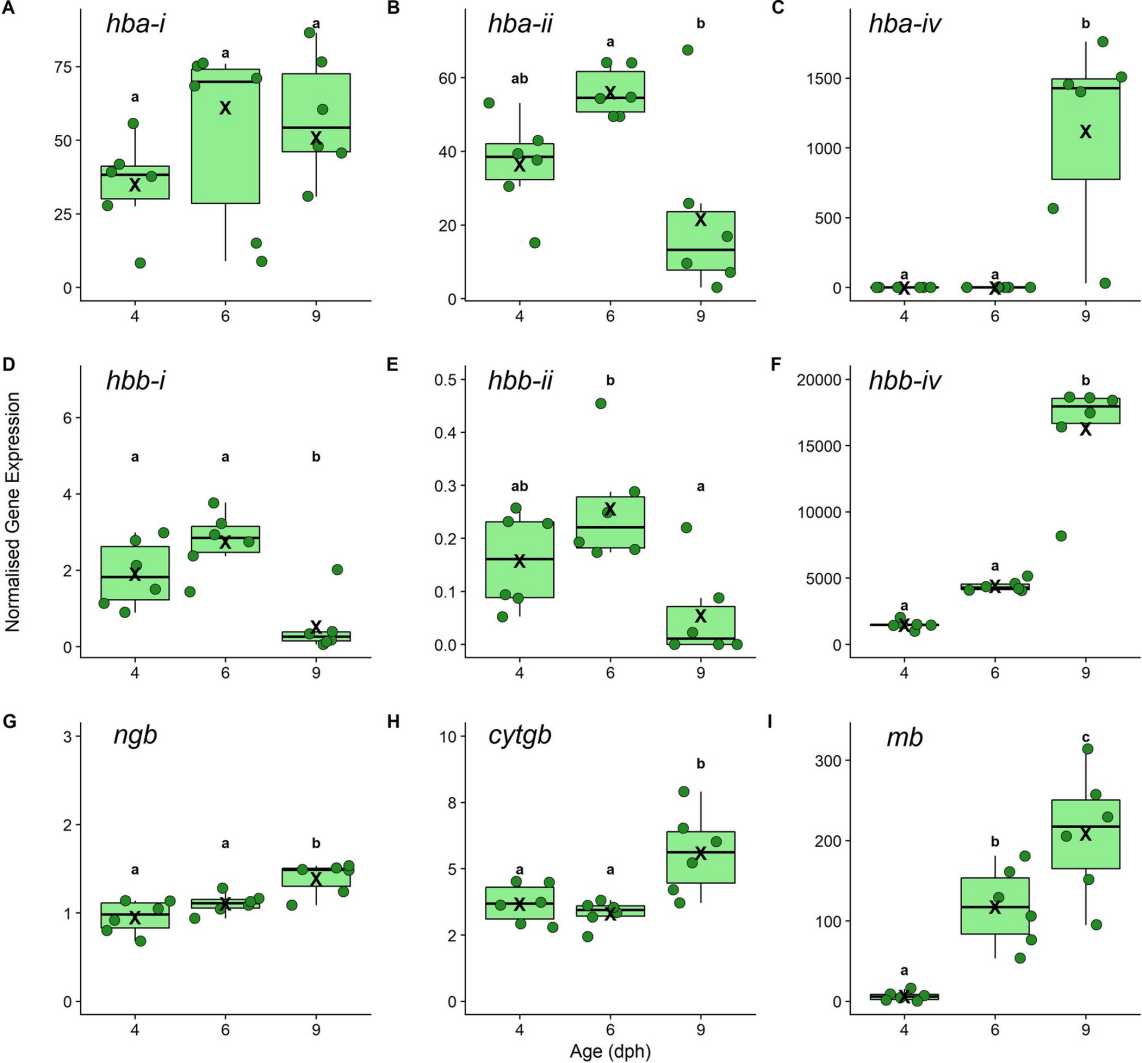
Normalised gene expression of (**A**) Hb subunit alpha paralog i (*hba-i*), (**B**) paralog ii (*hba-ii*), (**C**) paralog iv (*hba-iv*), (**D**) Hb subunit beta paralog i (*hbb-i*), (**E**) paralog ii (*hbb-ii*), and (**F**) paralog iv (*hbb-iv*), (**G**) neuroglobin (*ngb*), (**H**) cytoglobin paralog ii (*cytgb*), and (**I**) myoglobin (*mb*) measured in larval anemonefish (*Amphiprion melanopus*) aged 4, 6, and 9 dph. Each point represents normalised gene expression from an individual larva (*n =* 6 individuals per age). Boxplots show median and interquartile ranges, “X” indicates average gene expression for each day per gene, and different lowercase letters represent statistical differences in normalised gene expression between ages (LMs; **α** = 0.05). Ensemble gene IDs and data for all genes can be found in sheet 1 in S2 Data. The data underlying this figure can also be found in sheet 4 in S1 Data, and details on statistical outputs can be found in the supporting information S1 StatisticalOutput. dph, days post hatch; Hb, haemoglobin; LM, linear model.

To obtain expression patterns of the specific globin genes and to investigate overall changes in the transcriptome over early ontogeny (see below), we sequenced mRNA extracted from whole larvae and used the genome of a closely related species, *A*. *ocellaris*, for mapping and expression quantification. We extracted the normalised mRNA expression from the RNAseq dataset of the 15 identified globins in the *A*. *ocellaris* genome to determine whether gene expression changes around the onset of hypoxia tolerance (4 and 6 dph) and settlement (9 dph) (see S2 Data). These included several paralogous genes coding for Hb alpha (*hba*) and beta (*hbb*) subunits (i.e., the combined heterotetramer, Hb, binds oxygen in red blood cells), myoglobin (*mb*; oxygen storage and delivery into muscle tissue), cytoglobin (*cytgb;* oxygen storage in connective tissue), and neuroglobin (*ngb;* oxygen storage in neurons). For both the alpha and beta Hb subunits, there was a clear switch in expression from 4 dph to 9 dph (Fig 3A–3F), as detailed below.

Two hba gene paralogs, *hba-i* and *hba-ii*, increased slightly, but not significantly, from 4 to 6 dph (*hba-i*: *p* = 0.43; *hba-ii*: *p* = 0.12*)*, but *hba-ii* decreased at 9 dph (*hba-ii*: 4 dph versus 9 dph, LM *p* = 0.28, 6 dph versus 9 dph, LM *p* = 0.0057), whereas, *hba-i* remained statistically the same at 9 dph (4 dph versus 9 dph, LM *p* = 0.24, 6 dph versus 9 dph, LM *p* = 0.91) (Fig 3A and 3B). A third paralog, *hba-iv*, was expressed minimally at 4 and 6 dph but increased significantly at 9 dph (4 dph versus 9 dph, LM *p* = 0.0004, 6 dph versus 9 dph, LM *p* = 0.0004) (Fig 3C). Three hbb paralogs, *hbb-i*, *hbb-ii*, and *hbb-iv*, were present at 4 and 6 dph but lowly expressed; *hbb-i* and *hbb-ii* decreased in expression by 9 dph, albeit *hbb-ii* was not significantly different at 4 dph than 9 dph (Fig 3D–3F). The fourth paralog *hbb-iv* increased significantly in expression from 4 to 9 dph (*hbb-iv*: 4 dph versus 9 dph, LM *p* < 0.0001; 6 dph versus 9 dph, LM *p* < 0.0001) (Fig 3F). The genes coding for neuroglobin (*ngb*) (LM, *p* < 0.02 for both 4 dph and 6 dph versus 9 dph), cytoglobin (*cytgb*) (LM, *p* < 0.01 for both 4 dph and 6 dph versus 9 dph), and myoglobin (*mb*) (LM, *p* < 0.02 for both 4 dph and 9 dph versus 9 dph) all exhibited highest expressions at 9 dph compared to earlier larval stages (Fig 3G–3I).

### Changes in the transcriptome over early ontogeny

**Fig 4:**
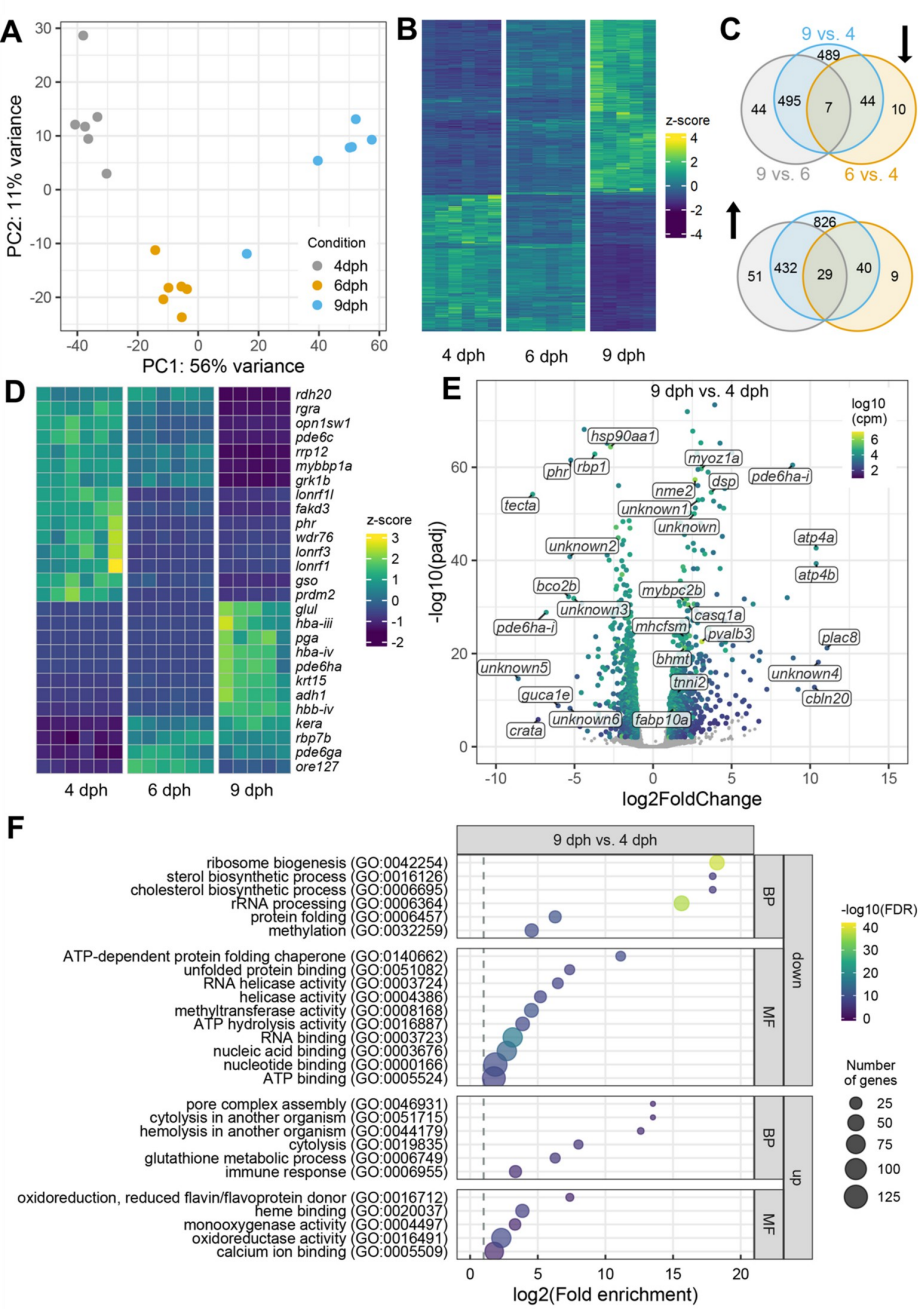
(**A**) Plot of PC1 and PC2 of variance-stabilised gene count data. There was one outlier in the 9-dph group, which was excluded from further analysis to increase the statistical power. Raw counts for all genes available in sheet 2 in S2 Data. (**B**) Z-score normalised heatmap of all DEGs (adjusted *p*-value < 0.05 and fold change > 1.5). Data available in sheet 3 in S2 Data. (**C**) Venn diagram for the up- and down-regulated genes. (**D**) Z-score normalised heatmap of the top 10 most significant genes in each comparison. Data available in sheet 4 in S2 Data. (**E**) Volcano plot for 9 dph larvae compared to 4 dph larvae. Data for top tags available in sheet 5 in S2 Data. Coloured symbols are significant DEGs. (**F**) GO enrichment analysis of DEGs in 9 dph larvae compared to 4 dph larvae. For clarity, only GO terms with FDR lower than 0.00005 and 0.0005 are shown for down- and up-regulated genes, respectively. Data available in sheet 6 in S2 Data. The complete lists of DEGs and statistical data for each comparison are available in sheets 7a, 7b, and 7c in S2 Data. BP, biological process; DEG, differentially expressed gene; dph, days post hatch; FDR, false discovery rate; GO, gene ontology; MF, molecular function; PC1, principal component 1; PC2, principal component 2.

Larval development is characterised by changes in anatomy and physiology and regulated by changes in gene expression. To capture this, we characterised changes in the transcriptome of cinnamon anemonefish more broadly, as larvae prepare to transition from pelagic to reef life. We expected changes to occur in expression for a variety of genes besides the globins described above. A principal component analysis including all genes (24,774) from the RNAseq dataset revealed a tight clustering of the data according to the sampling time points—i.e., with a clear separation between 4 dph, 6 dph, and 9 dph larvae (Fig 4A), except for one 9 dph individual that seemed closer to the 6 dph group (note: this individual was excluded from further analyses) and with 61% of the variation explained by the 2 first principle components. In total, the analyses identified 2,470 genes as differentially up- or down-regulated (i.e., adjusted *p*-value lower than 0.05 and fold change larger than 1.5) in either of the 3 time points compared to another (Fig 4B).

Most of the differentially expressed genes (DEGs) were found when comparing 9 dph to 4 dph and 6 dph (Fig 4C), with an overweight of up-regulated genes. Some genes were up- or down-regulated already at 6 dph when compared to 4 dph, but only a few genes were regulated at 6 dph only (see sheets 8a and 8b in S2 Data for these genes). The most down-regulated gene here was *mfsd4ab* (major facilitator superfamily domain containing 4A), which decreased 16 times, while gene expression of a putative protease-activated receptor, *f2r* (coagulation factor II (thrombin) receptor), was up-regulated 3 times. Likewise, there were few genes that were differentially regulated only at 9 dph (see sheets 9a and 9b in S2 Data). Here, *slc15a1a* (solute carrier family 15 member 1-like) decreased 4 times, while *cpa1* (carboxypeptidase A1-like) increased almost 3 times.

Among the topmost significant DEGs overall (Fig 4D), there were decreases at 9 dph in genes coding for opsins (*opn1sw1*; putative violet-sensitive opsin, *gso*; green-sensitive opsin) and an increase in an odorant receptor (*ore127*; odorant receptor, family E, subfamily 127, member 1). There were also decreases at 9 dph in some vision-related intracellular signalling proteins (*pde6c*; cone cGMP-specific 3’,5’-cyclic phosphodiesterase subunit alpha-like retinal cone, *rgra*; RPE-retinal G protein-coupled receptor-like, *rdh20*; retinol dehydrogenase 10-A-like, *grk1*; rhodopsin kinase-like), and increases in retinal cone and rod rhodopsin-sensitive cGMP 3’,5’-cyclic phosphodiesterase subunits (*pde6ha* and *pde6ga*). There were also changes in expression of *mybbp1a* (MYB binding protein 1a), which in humans is suggested to have a role for circadian rhythm, and *phr* (deoxyribodipyrimidine photo-lyase-like), which may be involved in UV radiation-induced DNA damage [20]. Lastly, several of the genes coding for Hb subunits as shown in Fig 3 were also among the most significant DEGs (*hba-iii*, *hba-iv*, and *hbb-iv*) (Fig 4D).

Looking more specifically at 9 dph compared to 4 dph (Fig 4E), some of the genes with highest increases in expression code for the alpha and beta subunit of the gastric H^+^/K^+^ ATPase pump (*atp4a* and *atp4b*) and cerebellin 20 (*cbln20*), a gene seemingly specific to teleosts, but a member of the family of cerebellins, which have a putative neuromodulatory function [21]. Several genes coding for proteins seemingly related to muscle function were also highly up-regulated (*mhcfsm*, myosin heavy chain, fast skeletal muscle-like; *mybpc2b*, myosin-binding protein C, fast-type-like; *myoz1a*, myozenin 1; *tnni2*, troponin I, fast skeletal muscle-like; *mylpf*, myosin light chain, phosphorylatable, fast skeletal muscle; *casq1a*, calsequestrin-1-like). Some of the most expressed genes were *nme2* (nucleoside diphosphate kinase B-like) and *pvalb3* (parvalbumin alpha-like), which both increased. A gene coding for heat shock protein 90, *hsp90aa1*, was also highly expressed but decreased significantly at 9 dph compared to 4 dph. A gene coding for alpha-tectorin, *tecta*, which in humans has a putative role in sensory perception of sound [20,22], was also highly expressed and significantly decreased. Lastly, there were significant increases and decreases in genes coding for proteins without similarity to any known proteins or function (*unknown1* to *unknown6*).

To investigate common functions between the 2,362 genes identified as differentially expressed at 9 dph compared to 4 dph, we carried out gene ontology (GO) enrichment analyses for down- and up-regulated genes separately (Fig 4F). The set of down-regulated genes was most significantly enriched for the GO terms “ribosome biogenesis” (GO:0042254, “rRNA processing” (GO:0006364) and “RNA binding” (GO:0003723), but also “methylation” (GO:0032259) and “methyltransferase activity” (GO:0008168). The latter could indicate that epigenetic regulation of transcription was taking place. The set of up-regulated genes was enriched for terms related to oxireductase activity (GO:0016491, GO:0004497, GO:0016712) and heme binding (GO:0020037) and may reflect the changes in oxygen-binding proteins, such as Hb.

## Discussion

Strong swimming capabilities, along with well-developed sensory systems and behaviours, are needed for pelagic larval reef fish to reach a coral reef after developing in the open ocean [3–5,8,23–25]. Without such performance, connectivity among populations may be highly limited, and low dispersal of larvae would result in genetic bottlenecks that may weaken the viability of future reef fish populations. Additionally, the physiological demands for tolerating hypoxic events during settlement are a critical, yet often overlooked factor for coral reef fish larvae to successfully transition between distinct pelagic and reef habitats. Our results show that the cinnamon anemonefish, in accordance with our hypothesis, has very high oxygen uptake rates, and this may contribute to supporting its fast swimming speeds and rapid development within a narrow 9-day window. Mass-specific oxygen uptake rates decreased midway through development, as predicted, and this decrease co-occurred with progressively increased tolerance to hypoxia and a switch in expression of Hb isoforms as well as increased gene expression of Mb, Ngb, and Cytgb. While the roles of some of these globins are debatable within the context of oxygen storage and transport for ectotherms, their expression is highest when hypoxia tolerance is also at its highest, suggesting that these globins may contribute to the development of low oxygen tolerance, even at later stages when swimming performance is also highest. While these results indicate a correlation, and further manipulative studies are necessary to confirm causation, they do fill critical knowledge gaps regarding the physiology of coral reef fishes during early life.

Most fish larvae, especially temperate species, require several months to successfully metamorphose into juveniles and recruit to adult populations [5,6]; anemonefish larvae grow and develop rapidly and metamorphose by about 9 dph. Here, we found that, across their entire pelagic stage, larvae of cinnamon anemonefish have among the highest mass-specific oxygen uptake rates (i.e., both SMR and MMR) of any teleost fish species studied to date [9]. Specifically for anemonefishes and direct developing fishes that are well developed upon hatch [26], high SMR may be required to support oxygen demands of development and growth of complex tissues (e.g., eyes and brain) [26–28]. Additionally, anemonefish, specifically cinnamon anemonefish, are capable of swimming—a highly energetically demanding activity—immediately upon hatching, which will add to their metabolic demands. Larvae were swimming slower than 3 BL s^−1^ at hatch but exceeded 10 BL s^−1^ approaching settlement—significantly faster than temperate species at similar life history stages [5,6]. Slight changes in swimming performance between our study and previous work on this species may be attributed to chamber design and duration of swimming protocol [29,30].

How oxygen uptake rates change with size and life history strategies has been debated for several decades [31]. The scaling exponents obtained here for mass-specific oxygen uptake rates (−0.8) differ from more commonly observed scaling exponents (−0.2 to −0.3). Thus, the mass-specific oxygen uptake rates in cinnamon anemonefish larvae decreased more with size (and hence age in this study) than would be expected if it was a simple scaling effect. This observation could have to do with the high metabolic activity of these larvae (i.e., high swimming capacity, growth rate, and degree of development at hatch) when compared with other similar size fishes. Indeed, in anemonefishes—and potentially reef fishes in general—life history may be optimised over evolutionary history to accommodate such metabolic performance [31] and demands to be aerobically fit to swim but also prepared for reef conditions. Hypoxia-tolerant fishes generally have lower oxygen uptake rates to reduce ATP (adenosine triphosphate) usage during low-oxygen conditions while still meeting metabolic demands [9]. It is possible that the high oxygen demands in 1 to 4 dph larvae may be too high to support metabolic functions under hypoxic conditions on the reef; larvae aged 4 dph had the poorest hypoxia tolerance when compared to larvae measured at later stages. Therefore, the measured decrease in oxygen uptake rates between 4 dph and 6 dph, coupled with progressive hypoxia tolerance during this period, may indicate an “optimization” for improved physiological performance on the reef, but further studies are needed to determine causation. We hypothesise that the lack of further change in mass-specific oxygen uptake rates from 6 to 9 dph, despite significant increases in body size, swimming performance, and hypoxia tolerance within these ages (especially at 9 dph), may suggest that these respiration rates are sufficient to support these functions.

Previous studies investigating gene expression during larval fish ontogeny used specific developmental milestones (e.g., onset of metamorphosis and switch from endogenous to exogenous feeding) and formation of structures (e.g., muscle, fins, eyes, and digestive system) as markers to indicate when to collect samples [32–36]. Instead of morphology or defined developmental stages, we used a physiological marker (i.e., decreases in oxygen uptake rates) to indicate where we might expect changes in expression for genes associated with oxygen transport. Indeed, our data revealed several changes in the transcriptome of larval cinnamon anemonefish from 4 dph to 9 dph (i.e., at the time when oxygen uptake rates and hypoxia tolerance also changed). These changes included genes that may not be associated with hypoxia tolerance *per se* but changed during development as fish approached settlement; some of these gene products were most similar to proteins with roles in vision, digestion, and olfactory systems. Other genes can be speculated to be more closely associated with the fact that the larvae are transitioning to a low-oxygen environment. For example, alcohol dehydrogenase (*adh1*) was also up-regulated at 9 dph, and animals have been found to exhibit a correlation between alcohol dehydrogenase enzyme activity and the ethanol content in their food [37,38]. Therefore, the increase in *adh1* expression that was observed at 9 dph could suggest that the larvae are getting prepared to ingest food that may contain ethanol, which is likely to be introduced into the food chain by fermenting microorganisms during nocturnal hypoxia on the reef. However, among the most significant changes in gene expression were those associated with Hb subunit composition, which coincided with an increased hypoxia tolerance that we speculate may support the transition between pelagic to reef habitats.

The oxygen affinity of the blood is partly determined by the properties of the Hb isoforms (i.e., the tetramers) that are expressed at any given time and the affinity of the isoforms are in turn determined by the properties of the individual subunits. Hence, small changes in Hb subunit composition can greatly affect the oxygen binding affinity of Hb [39,40]. However, it is important to note that there are several other modulators of oxygen binding affinity, such as organo-phosphates (e.g., ATP and guanosine triphosphate (GTP)), which may also contribute to shifts in Hb oxygen affinity over ontogeny [39,41]. Fishes are also capable of shifting Hb oxygen affinity in response to changes in environmental conditions [42]. For example, at hatch, salmon fry live under the gravel for some time where they must be hypoxia tolerant; indeed, they possess Hbs that have a high binding affinity, which is thought to prevent hypoxemia [14,16]. This binding affinity shifts when fry emerge from the gravel as highly active smolts, thus allowing for greater release of oxygen to tissues needed to support swimming [14,16,43,44]. As they develop into juvenile phases, zebrafish and gilthead seabream (*Sparus auratus*) retain some embryonic Hbs [42,45,46], which have higher oxygen binding affinities and are used to supply tissues with oxygen in case of unexpected hypoxia or intense swimming [42,45,46]. Cinnamon anemonefish are predictably exhibiting the opposite pattern to salmon, lowering oxygen demands midway through their pelagic larval stage, possibly to support hypoxia tolerance, which would prepare them for the oncoming hypoxia challenges associated with settlement onto a reef, 3 to 4 developmental days into the future. Hb genes *hba-i*, *hbb-i*, *hba-ii*, and *hbb-ii* were more dominantly expressed at these ages (4 dph and 6 dph) when the fish are most sensitive to hypoxia. We hypothesise that these early-expressed (i.e., 1 dph to 4 dph) Hb subunits have low oxygen affinity to ensure adequate O_2_ off-loading at the tissues, thereby supporting growth and development as well as high swimming capacity [42]. Upon settlement (9 dph), *hbb-i* and *hbb-ii* decreased in expression, and a switch in dominant Hb subunits occurred with the increased expression of *hba-iv* and *hbb-iv*. We expect that these late-expressed Hb subunits have a higher oxygen affinity, as the increased expression of these subunits parallels the increase in whole-organism hypoxia tolerance, but more detailed experiments are needed to investigate these hypotheses.

At settlement (9 dph), Mb, Ngb, and Cytgb gene expression also increased. These 3 proteins are known to have high oxygen affinity in other species, and while the role of these proteins in oxygen storage and transport has been debated for ectotherms, some studies have demonstrated their function for hypoxia tolerance (i.e., oxygen storage for tissues during low-oxygen conditions) in fishes [47]. Mb is found in high concentrations in the skeletal muscle and heart of diving mammals (seals and whales) and mammals living at high altitudes to store oxygen under hypoxia and to reduce Reactive Oxygen Species (ROS) accumulation and protect cells during reoxygenation after hypoxia [48,49]. The role of Mb as an oxygen store is debated for fishes, as oxygen availability varies greatly among aquatic habitats, and due to the variety of lifestyles (e.g., benthic to pelagic “athlete”), there may not be a widespread requirement for such oxygen stores [48]. However, it has been proposed that, in hypoxia-tolerant fishes such as carp, Mb functions as cellular protection in low-oxygen environments [18,48]. Additionally, zebrafish exposed to chronic hypoxia for several weeks showed a 138% increase in Mb expression in highly oxidative muscle fibres, and it was proposed that it facilitated oxygen supplied to mitochondria [50]. The specific role of Ngb in fishes has been debated since its discovery in fish brains [51] but has been proposed to supply oxygen to highly oxygen-demanding neural tissue under hypoxic conditions [51–53]. Cytgb has been found in the connective tissues in fishes and also in high concentrations in zebrafish red blood cells; it may serve as an alternative oxygen transporter in low-oxygen conditions [19,47,53]. While our study cannot elucidate the exact function of these globins *per se*, there may be a correlation between their increased expression and progressive hypoxia tolerance in the cinnamon anemonefish over early development that suggest their roles for retaining oxygen in tissues under low-oxygen conditions and possibly during intense exercise as well. Further work involving gene silencing could demonstrate whether hypoxia tolerance and possibly swimming performance change without these genes being expressed.

The very high oxygen demands of larval anemonefish may have notable trade-offs if larvae encounter rapid or unpredictable changes in environmental conditions. Until this study, the AAS (i.e., the difference between MMR and SMR) of tropical reef fish larvae was unknown [54]. The AAS of coral reef fish larvae has been assumed to be low, due to predictably high metabolic costs associated with growth and development (high SMR), and because it was assumed their metabolic rate could not increase much higher above their SMR [55,56]. Our respiratory profile measuring aerobic scope (AS) during swimming over the entire larval duration partially supports these assumptions. We show that, while SMR is high, swimming larvae also have a relatively high MMR and consequently have a high AAS. A high AAS complements the early life history of reef fishes that need to grow and develop fast within a narrow development window (i.e., high SMR) and to be able to support the high swimming performance (i.e., high MMR) that has presumably been selected for to reach a suitable reef. However, the relatively low FAS of 2.2 on average (representing how many-fold an organism can increase its oxygen uptake rate above rest) may suggest that anemonefish larvae have a limited capacity to tackle a major environmental challenge such as elevated temperature [57]. Assuming a temperature coefficient (Q_10_) of 2 would mean 1.5 times higher SMR with a 5 degree increase in temperature, which would then take up a large proportion of the AS in these larvae, despite the high AAS. This could be attributed to the relatively narrow thermal window tropical species are exposed to on a seasonal basis and that they live near the edge of their thermal limits [58]. Previous work has demonstrated that small increases (1°C to 3°C) in temperature outside of their normal thermal regime [59,60] and increased turbidity [61] significantly reduce the AS of coral reef fishes. This contrasts with temperate fishes that have a wider thermal breadth, as they cope with greater seasonal variations, and may therefore be more resilient to environmental change. Reduced aerobic capacity in response to environmental stress could result in low growth, reduced developmental rates, and less energy available for swimming, therefore potentially reducing dispersal and connectivity among reef fish populations. Thus, the high metabolic activity (i.e., high SMR and MMR) and low FAS of tropical coral reef fish larvae may make them more susceptible to ocean warming and marine heatwaves.

## Conclusions

Since the early 1990s, it has become apparent that high swimming performance as a larva is an ecologically relevant characteristic of coral reef fish life history, as connectivity among reef fish meta-populations would not be possible without such capabilities [4,7,24,25,62–68]. Reef fishes must undergo significant changes to their metabolic machinery, as their life history simultaneously demands high swimming performance and developing hypoxia tolerance prior to settling onto a reef. Our integrative approach, while correlative in nature, shows changes in oxygen uptake and gene expression of globins that we suggest may contribute to successful transition from pelagic to reef habitats. Without such flexibility in physiological traits, dispersal and recruitment would likely be spatially limited, which could increase the potential for genetic bottlenecks. While this developmental strategy may come with trade-offs if larvae experience unexpected environmental stressors, developing from larva to juvenile within a narrow time window may not be possible without such high oxygen demands. To determine how much of their energy is invested in responding to environmental stress, we encourage future studies to integrate multiple stressors (i.e., temperature, CO_2_, hypoxia, turbidity, etc.) to larval fish swimming respirometry experiments to investigate the limits of their adaptive capacity during this crucial developmental transition. Additionally, experimentally manipulating the expression of genes coding for Mb, Ngb, and Cytgb may provide a better understanding of how these globins function in coral reef fishes, and obviously it is of interest to investigate the functional properties (e.g., oxygen binding affinity) of the early and late expressed Hb isoforms. Our study involved a single species, but given that most reef fishes face similar ontogenetic challenges, it is likely that they also exhibit similar changes in oxygen demands and gene expression patterns for oxygen transport proteins.

## Methods

All larvae rearing, fish husbandry, and experimentation occurred at James Cook University at the Marine and Aquaculture Research Facilities Unit (MARFU). All husbandry and experimentation protocols were approved by James Cook University’s animal ethics committee (approval #A2425).

### Choice of study species

Most coral reef fishes have not been successfully bred in captivity, and newly hatched and very early larval stages are not typically captured (e.g., via light traps) in the wild, which precludes most studies where a fine-scale assessment at each life stage is required. Using the cinnamon anemonefish (*Amphiprion melanopus*) as a contemporary model/representative species works well in this case, given that anemonefishes represent some of the few species that have been successfully bred in captivity where a high proportion of larvae hatch and survive to metamorphosis to be reared and bred for subsequent generations. Because most coral reef fishes have a bipartite life history where parents spawn in the reef, eggs or larvae are carried off the reef into the open ocean by currents, and then larvae develop in the pelagic environment before swimming back to the reef to metamorphose and settle, our findings can be a starting point for other species and can provide the first mechanistic underpinnings as to how reef fishes transition from pelagic to reef habitats during early life history.

### Husbandry of study species

Adult breeding pairs of the cinnamon anemonefish were originally captured on the Great Barrier Reef in 2015 by commercial divers (Cairns Marine) and established at MARFU for long-term experimentation. For this study, adult pairs were maintained in 60 L flow-through outdoor aquaria at MARFU under natural conditions throughout the duration of the experiment (temperature = 28°C, salinity = 33 ppt, natural photoperiod). Adults were fed twice daily using pellet food (NRD G12 Inve Aquaculture, Salt Lake City, USA). Within each tank, there was half a terra-cotta pot for shelter and a place for adults to lay their eggs. Tanks were cleaned weekly to maintain water quality (ammonia below 0.04 ppm; nitrites below 0.75 ppm and nitrates between 10 and 40 ppm).

Adults generally lay eggs fortnightly, which typically hatch about 7 to 8 days later. On the day prior to predicted hatching, the terra-cotta pot was removed from the adult tank, promptly replaced with a blank pot, and transported in water to a 100-L flow-through larval rearing tank, which was in a separate, indoor room. In the larval rearing tank, water quality conditions were maintained as above aside from photoperiod (13 h:11 h, light:dark), and an air stone was placed under the eggs to simulate parents aerating the eggs and to promote hatching. From hatching to 5 dph (0 to 5 dph), larvae were fed rotifers (*Brachionus* sp.) at a concentration of 20 individuals ml^−1^. From 0 to 3 dph, 3 ml of algal paste (*Nannochloropsis* sp.) was also added to the tanks to feed rotifers and shelter larvae from light stress. From 3 to 9 dph, larvae were fed freshly hatched *Artemia* sp. nauplii, ad libitum. During feeding, water was switched off for 1 h to prevent food and algae from being removed from the tank, thus allowing larvae to have adequate time to feed after which water was switched back on to flush the system and maintain water quality.

### Swimming respirometer

Each fish was swum using a custom-built, glass, Blazka-style swimming respirometry chamber (volume (*V*) = 125 ml; length (*L*) = 14.5 cm; diameter (⌀) = 2.7 cm), which permits a simultaneous measure of oxygen uptake rates (*Ṁ*O_2_) while an individual swims at any given speed. The swimming respirometry chamber was calibrated prior to experimentation using a high-speed camera (Casio Exilim High Speed Camera) and passive particles. To reduce the volume of the respirometry chamber, thus providing more accurate *Ṁ*O_2_ measurements for the size of animals swum, an insert (⌀ = 2.6 cm, *L* = 8.5 cm) was placed in the respirometry chamber to create a smaller working section (*V* = 38 ml, *L* = 4.5 cm, ⌀ = 2.7 cm), which fit a smaller chamber (*V* = 3.5 ml, *L* = 2 cm, ⌀ = 1.5 cm) where the individual fish swum. This smaller chamber was fitted with a flow straightener (capillary tubes; ⌀ = 1.1 mm, *L* = 40 mm) to mitigate microturbulent flow and a downstream mesh barrier (mesh ⌀ = 0.415 mm) to prevent the individual fish from being sucked into the propeller. The swimming chamber was large enough for an individual fish to swim in any direction comfortably (i.e., to mitigate enclosure stress) while prevented blocking effects (< 5% of the chamber’s cross-section), which would alter the flow within the chamber. An external flush pump was used to deliver clean, fully aerated seawater (temperature, *T* = 28°C; total pressure, *P* = 758 mm Hg; salinity, *S* = 33 ppt, oxygen partial pressure, PO_2_ = 152.86 mm Hg, dissolved oxygen, DO_2_ = approximately 6.45 mg L^−1^) to the system in between measurement periods (see Experimental protocol for details). Oxygen uptake rates (mg O_2_ L^−1^) and *T* (°C) were simultaneously measured (oxygen probe: OXROB3 Robust Oxygen Probe, PyroScience, Achen Germany; *T* sensor: TSUB36 Shielded submersible temperature sensor, Pyroscience, Achen, Germany). Oxygen probes were calibrated to 100% of air saturation using fully aerated seawater (*T* = 28°C, *S* = 33 ppt, *P* = 1,011 hpa, PO_2_ = 153 mm Hg, DO = 6.45 mg O_2_ L^−1^) and to 0% oxygen saturation (*T* = 28°C, *S* = 33 ppt, *P* = 1,011 hpa, DO = 0 mg O_2_ L^−1^) using sodium sulphite (Na_2_SO_3_; UNIVAR Analytical Reagent, Ajax Finechem, New South Wales, Australia). Oxygen and temperature probes were connected to a Firesting 4-channel optical oxygen meter (Pyroscience, Achen Germany) that constantly measured both of these variables throughout each experiment (1 Hz, or s^−1^). The Firesting optical oxygen meter has integrated atmospheric pressure sensors for automatic pressure compensation of the oxygen measurements. Water temperature was maintained at experimental conditions (*T* = 28°C), even at high water velocities, by means of a temperature jacket (*V* = 85 ml, *L* = 6.5 cm). Prior to and following all swimming trials, all components of the swimming respirometry chamber were washed in a 10% bleach solution to eliminate any microbial activity from the system.

### Experimental protocol

The experiment was designed to measure daily ontogenetic changes in oxygen uptake rates (*Ṁ*O_2_) during periods of activity over the entire larval phase of the anemonefish. Because we measured *Ṁ*O_2_ on active larvae, we can also estimate MMR (estimate of primarily oxygen demands of an ectotherm under intense exercise), SMR (estimates of oxygen demands of an ectotherm at rest), and calculate AS (animal’s capacity to increase their oxygen uptake rates above rest) from each swimming experiment (see below for further details). Each developmental age was exposed to the same protocol. Experiments were conducted daily on larvae from time at hatch (0 dph) until settlement (9 dph) (*n =* 8 to 10 larvae per developmental age). Since this study relies heavily on ontogenetic changes in metabolic rate, for consistency, all larvae used in this experiment came from the same parent anemonefish breeding pair to mitigate parental effects, all larvae were reared in the same rearing aquaria to eliminate potential tank effects, and all larvae were individually swum using the same swimming respirometry chamber. Multiple clutches were used to achieve the sample size for each developmental age, but breeding pair consistency aligned with the goal of mitigating any potential differences in physiology between offspring of different breeding pairs. Occasionally, only 50% to 60% of a clutch would hatch with remaining larvae hatching 1 to 3 days later. These split clutches were removed from the rearing tank, and the pot was placed in a sperate rearing tank adjacent to the other tank, using the same water supply (i.e., same conditions between tanks) until the remaining larvae hatched. This prevented mixing of ages (it is difficult to tell ages apart), which would influence results, and the lag between ages allowed us to back-fill to previous days to maximise the use of the clutch.

All experiments were performed in a dark experimental room at MARFU, which was separate from the larval rearing room to prevent external stimuli from influencing the experiment; however, a red headlamp (600 lumens, Ledlenser MH10; Ledlenser Australia, New South Wales) was used during the experiments. Prior to each individual experiment, background respiration (i.e., microbial oxygen uptake rates) was measured for 10 min [69]. To mitigate microbial respiration, the seawater (28°C) used for all experiments was passed through an ultraviolet light filter (Blagdon Pro 24W ultraviolet clarifier, Dreative Pumps, South Australia, Australia). Larvae were fasted for at least 12 to 15 h before experimentation (i.e., not fed overnight) to ensure they were in a post-absorptive state and there would be no influence of digestion (i.e., specific dynamic action) on oxygen uptake rates [57]. Then, an individual larva was gently removed from the rearing tank, placed in a black covered bucket, and gently transported to the experimental room (< 2-min time frame). The individual larva was then placed into the swimming respirometry chamber by gently pouring it in and then quickly positioning it into the working section of the swimming respirometry chamber. This was performed in a separate tank (“preparation tank”), so the entire swimming respirometry chamber could be sealed underwater to prevent air bubbles from building up in the system. The swimming respirometry chamber was then gently removed from the “preparation tank,” and then the motor was attached. Each individual fish was allowed to habituate to the chamber and recover from handling/transport stress for 60 min, under constant, gentle flow conditions at a water velocity equivalent to 1 body length per second (BL s^−1^; a subsample of individuals was measured per species [total length; snout to tip of caudal fin] to provide an overall estimate for BL).

Individual larvae underwent a stepped velocity test, post-habituation, to measure critical swimming speed (*U*_*crit*_), a test designed to estimate the (primarily) aerobic capacity of fishes during swimming [70,71]. Every 20 min, the water velocity in the chamber was increased by 1 BL s^−1^ until the individual fish fatigued, as indicated by impingement on the downstream barrier [70,71]. Critical swimming speed was calculated using the following formula:

$$U_{\text{crit}\;}\left({\text{cms}}^{-1}\right)={\text{V}}_{\text{f}}+(\text{T}/\text{t})*{\text{V}}_{\text{i}}$$

where V_f_ is the penultimate speed (cm s^−1^), T is the time swum at the fatigue speed, t is the time interval (20 min), and V_i_ is the velocity increment (approximately 1 BL s^−1^) [70]. We converted *U*_*crit*_ to BL s^−1^ by dividing each individual’s *U*_*crit*_ by its BL (cm). At each swimming speed, *Ṁ*O_2_ was measured using intermittent flow respirometry, consisting of a 20-min measurement period (i.e., the time interval portion of the *U*_*crit*_ protocol), followed by a 3-min flush period (i.e., to replenish the swimming chamber with clean, fully aerated seawater, which lasted until the water velocity increases to the next speed) [69]. The flush period was long enough for oxygen levels within the swimming chamber to be replenished to 100% of air saturation (DO = 6.45 mg O_2_ L^−1^). However, oxygen within the swimming chamber was never allowed to fall below 90% air saturation to prevent oxygen uptake rates of the fish from being influenced by hypoxia [69]. Upon completion of a *U*_*crit*_ protocol, the fish was removed from the respirometry chamber, killed in an ice bath (so the larva could be weighed), and background respiration was measured for 10 min to account for accumulated microbial activity introduced by the fish.

Text files from the Firesting were imported and analysed in LabChart (ver 8, AD instruments, New South Wales, Australia) to calculate *Ṁ*O_2_ at each swimming speed. The oxygen uptake rate (*Ṁ*O_2_) at each 20-min interval was calculated as follows:

$$\dot{M}{\text{O}}_{2}\left({\text{mg}\;\text{O}}_{2}\;{\text{g}}^{-1}{\text{h}}^{-1}\right)=\text{S}\cdot {\text{V}}_{\text{resp}}{\text{M}}^{-1}$$

where S is slope of the linear regression during the measurement period (mg O_2_ s^−1^), V_resp_ is the volume of the respirometry chamber (minus the fish), and M is the mass of the individual fish (g) [69]. Commonly, mass in kilograms is used to standardise mass-specific MO_2_; however, for cleanliness of the figures (reduce the size of the numbers in the y-axis), we used body mass in grams. Background respiration was subtracted from each value of *Ṁ*O_2_. For each individual swimming experiment (i.e., for each individual larva per day), each *Ṁ*O_2_ value was plotted against each swimming speed (cm s^−1^). The appropriate linear regression (1) or power curve (2) was fit through this relationship:

R(u)=a+bu (1)

$$\text{R}(\text{u})=\text{a}+{\text{cu}}^{\text{b}}$$ (2)

where R(u) is an estimate of mass-specific oxygen uptake (mg O_2_ g^−1^ h^−1^) at any given speed (u; cm s^−1^), a is the y-intercept, b is the slope of the equation (linear regression; Eq 1) or scaling exponent (power curve; Eq 2), and c is an estimated parameter [72,73]. If a line could not be fitted through the points (e.g., line went through the negative y-axis), then the fish was omitted from *Ṁ*O_2_ analysis but was still used for *U*_*crit*_. It should be noted that fish at hatch (0 dph) did not have enough data points (swimming speeds) for accurate measures of SMR or MMR, but *U*_*crit*_ could be calculated since they swam at a low speed during the habituation period. The y-intercept of this relationship between *Ṁ*O_2_ and swimming speed provides an estimate of the fish’s SMR (mg O_2_ g^−1^ h^−1^), which is an estimate of the basic metabolic functions of the animal at rest (u = 0 cm s^−1^) [72]. The maximum *Ṁ*O_2_ value when the individual fish fatigued is an estimate of MMR (mg O_2_ g ^−1^ h^−1^), which is also an estimate of the maximum sustainable (i.e., aerobic) swimming speed [69]. AS (mg O_2_ g^−1^ h^−1^) was calculated by subtracting SMR from MMR and represents the total amount of oxygen available to the fish to perform aerobically driven tasks (swimming, finding food, avoiding predators, etc.) beyond basic maintenance [69]. AS was presented as both absolute scope (AAS = MMR − SMR) and factorial aerobic scope (FAS = SMR / MMR) for each developmental age, with the latter showing the fold increase between rest and maximum *Ṁ*O_2_ [57].

### Hypoxia tolerance

Hypoxia tolerance experiments were performed on larvae aged 4, 6, and 9 dph. These ages correspond with ontogenetic changes in *Ṁ*O_2_ (see Results). Hypoxia experiments involve placing an individual larva into a chamber (1.5 ml glass vial) and allowing it to naturally reduce oxygen within the chamber until the animal loses equilibrium [12]. An oxygen probe (oxb430 bare fiber oxygen microsensor; Pyroscience, Aachen, Germany) was affixed through the lid of the chamber, sealed into the lid using silicone, and placed near the bottom of the chamber where the larvae typically stayed. Oxygen probes were connected to a Firesting 4-channel optical oxygen meter (Pyroscience, Achen Germany), which measured the change in dissolved oxygen levels in the chamber as the fish respired. Because attempts to mechanically mix the chamber (e.g., using magnetic stir bar) resulted in too much turbulence in the chamber and inevitable death of the larva, mixing was solely reliant on movement of the larva.

Prior to the start of each hypoxia experiment (i.e., first trial of the day and between trials), all equipment was cleaned with a 10% bleach solution to eliminate microbial activity that would influence measured oxygen uptake rates. The chambers were submerged in water with a temperature of 28°C and fully saturated with O_2_, i.e., 100% of air saturation [PO_2_ (partial pressure) = 153 mm Hg and DO (dissolved O_2_) = 6.45 mg O_2_ L^−1^]. Then, individual larvae were gently placed into each chamber (4 chambers total; 1 larva per chamber); the chambers were sealed underwater and gently place into a water bath (28°C, larval rearing temperature, replenished between trials), which was covered to mitigate external stimuli. Larvae were visually checked every 2 to 5 min using a red light. The endpoint of each hypoxia experiment was LOE, which is a behavioural response to low oxygen levels that represents “ecological death” [12]. If a larva exhibited LOE for 10 to 15 s, the water oxygen level (% of air saturation) was recorded, and the chamber was gently removed from the water bath in a manner to not disturb the other chambers. Then, the larvae were killed in an ice bath; body mass (g) was recorded.

### RNA extraction and sequencing

A subset of 60 larvae from 4, 6, and 9 dph groups were killed as above and preserved in individual 1.5 ml vials containing RNAlater (Sigma, Germany) and stored at −80°C until RNA extractions were performed. These larvae were living under fully saturated oxygen conditions and had not experienced hypoxic conditions prior to sampling. These developmental days were selected based on ontogenetic changes in *Ṁ*O_2_ and hypoxia tolerance that could be linked to changes in expression patterns of genes associated with oxygen transport. We performed a trial extraction on the smallest sized larvae (4 dph) using the RNA extraction procedure (described below) with 1, 2, or 3 larvae pooled per replicate and determined that 1 larva was sufficient to extract enough RNA. In total, RNA was therefore extracted from 6 larvae for each developmental age (*n =* 18 total).

Using sterilised forceps, the RNAlater preserved larvae were removed from their individual vials, dabbed on aluminium foil to remove excess RNAlater, and subsequently added to a powerbead tube (ceramic 2.8 mm beads; Qiagen, Germany). A 600-μl aliquot of a DTT/RLT mix (containing 20 μl 2M Dithiothreitol [DTT; Sigma, Germany] and 1 ml of RLT buffer [lysis buffer; Quigen, Germany]) was then added into each PowerBead tube. The samples were homogenised in a bead beater (Benchmark Scientific Beadbug D1030 Microtube Homogenizer; Benchmark Scientific, USA) in two 45-s cycles at 5.5 to 6 m s^−1^ with 30 s in between cycles. The samples were then centrifuged (Mikro 185 centrifuge, Labgear, Australia) at 18,000*g* for 3 min. The resulting supernatant (hereafter, lysate) from each tube was then decanted into individual sterile, RNAse-free 1.5 ml tubes. Approximately 550 μl of 70% ethanol was added to each of the tubes containing lysate and mixed via pipette. From each tube, 700 μl of lysate was loaded onto an individual RNeasy column (RNeasy Mini kit, Qiagen, Germany) and centrifuged at 8,000*g* for 15 s. The flow-through was discarded. Then, the remaining sample from each tube was loaded onto their respective RNeasy column and centrifuged at 8,000*g* for 15 s, subsequently discarding the flow-through. To each RNeasy column, 350 μl of RW1 (wash buffer; RNeasy mini kit; Qiagen, Germany) was added and centrifuged at 8,000*g* for 15 s, subsequently discarding the flow-through. Then, 10 μl of DNase stock—which was prepared beforehand by dissolving lyophilised DNase (1500k units) in 550 μl of RNase-free water that had been divided into single-use aliquots and stored at −20°C until use—was added to 70 μl or RDD buffer (RNeasy mini kit; Qiagen, Germany) and mixed gently by pipetting. Exactly 80 μl of DNase/RDD buffer mix was added to each RNeasy column filter and allowed to incubate at room temperature for 15 min. Then, 350 μl of RW1 was added to each RNeasy column and centrifuged for 15 s, subsequently discarding the flow-through. Then, 500 μl of RPE buffer (wash buffer; RNeasy mini kit; Qiagen, Germany) was added to each column and centrifuged at 8,000*g* for 15 s, subsequently discarding the flow-through. Another 500 μl of RPE buffer was added to each column and centrifuged at 8,000*g* for 2 min. Afterwards, each RNeasy column was placed on a new collection tube and centrifuged at 18,000*g* for 1 min. Then, each RNeasy column was placed onto a clean labelled 1.5 ml tube; 50 μl of RNase-free water was directly added to each column and centrifuged at 8,000*g* for 1 min. This step was repeated to acquire 100 μl elutes. Elutes were stored at −80°C until shipped for sequencing. Samples were shipped on dry ice, and then sequencing was performed by the Australian Genome Research Facility (AGRF) in Melbourne. From the total RNA provided, poly(A) tail selected mRNA libraries were prepared using the Illumina Stranded TruSeq kit. The 18 samples were sequenced on 1 lane NovaSeq6000 SP with 300 cycles (150 bp PE), yielding 510,893,644 paired reads in total (per sample mean ± SD = 28,382,980 ± 5,130,537).

### Bioinformatics

Sequencing data were analysed on resources provided by Sigma2—the National Infrastructure for High Performance Computing and Data Storage in Norway. The raw sequence reads were trimmed using Trimgalore (0.6.2; Babraham Bioinformatics group; https://www.bioinformatics.babraham.ac.uk/projects/trim_galore/) with default parameters except for minimum length, which was set to 40 bp, and quality score, which was set to 20. The trimmed reads were quality checked using FastQC (0.11.8; Babraham Bioinformatics group; https://www.bioinformatics.babraham.ac.uk/projects/fastqc/) [74]. The trimmed reads were then aligned to the genome of the closely related ocellaris clownfish (*A*. *ocellaris*) using STAR (2.7.6a; [75]) in two-pass mode for discovery of novel splice junctions. The genome index was first prepared using genome and annotation files downloaded from Ensembl (release 107; [76]) with default parameters and—sjdbOverhang 149. Several trial runs were performed using different parameters to identify the command yielding an acceptable alignment rate, taking into consideration that the genome was from a closely related species and that reads were long. The final parameters used were as follows:

--twopassMode Basic
--peOverlapNbasesMin 12
--alignSJoverhangMin 10
--alignSJDBoverhangMin 10
--alignMatesGapMax 100000
--alignIntronMax 100000
--alignSJstitchMismatchNmax 1–1 1 1
--alignSplicedMateMapLmin 30
--alignInsertionFlush Right
--chimSegmentMin 12
--chimJunctionOverhangMin 8
--chimOutJunctionFormat 1
--chimMultimapScoreRange 3
--chimScoreJunctionNonGTAG -4
--chimMultimapNmax 20
--chimNonchimScoreDropMin 10
--outSAMattrRGline ID:GRPundef
--quantMode GeneCounts
--outSAMtype BAM SortedByCoordinate
--limitBAMsortRAM 3000000000
--outBAMsortingThreadN 5
--outSAMattributes All

The average alignment rate across samples of uniquely mapped reads was 76.1 ± 1.5%. Counting for downstream expression analysis was performed using the featureCounts tool from Subread (2.0.1; [77]) treating alignments as paired and reverse-stranded (-p -s 2), not counting chimeric alignments (-C), counting at the feature level exon (-t exon) but summarising at the meta-feature level gene (-g gene_id). Multi-mapping and multi-overlapping reads were discarded, but singletons (i.e., only 1 read in a pair had a valid alignment) were allowed. Of the alignments output by STAR, the average assignment rate across samples and included for counting by FeatureCount was 70.6 ± 0.8%.

Analysis and plotting of gene expression data were done in R (4.2.1). Raw count data (data available in sheet 2 in S2 Data) were used as input for identification of DEGs, but for plotting, data were normalised using the gene length corrected trimmed mean of M-values (GeTMM; [78]), which first correct for differences in the gene length (estimated as the merged exon length) and then accounts for differences in library size and composition across samples using the TMM normalisation function in the EdgeR package (3.30.3; [79]), which first correct for differences in the gene length (estimated as the merged exon length) and then accounts for differences in library size and composition across samples. For identification of DEGs, we used the DESeq2 package (v. 1.36; [80]) on the raw counts, setting the significance (parameter “alpha”) to < 0.05 and log-fold change threshold to 0.585 (parameter “lfcThreshold”; corresponding to a fold-change of 1.5), and using the lfcshrink() function with method “apeglm” [81] to obtain log2foldchange and its error estimate. The same criteria were used to filter the result to obtain a set of DEGs for each of the 3 comparisons. The R package VennDiagram (1.7.3; [82]) was used to generate Venn diagrams comparing numbers of up- and down-regulated genes that were unique or shared between different comparisons. The R package ComplexHeatmap (2.12.0; [83]) was used to generate a heatmap of all the identified DEGs as well as the top 10 most significant genes from each of the 3 comparisons. The R package ggplot2 (ver 3.3.6) was used to generate volcanoplot, PCA plot, and visualisation of GO enrichment analysis. The GO enrichment analysis was done using the package goseq (1.48; [84]). The package biomaRt (v. 2.52.0; [85]) was used to obtain GO terms for the gene IDs of *A*. *ocellaris*.

To further examine and compare the expression of specific globin paralogous genes across age groups, we searched for globin genes in the Ensembl genome browser for *A*. *ocellaris* (107). This search gave 15 hits, of which some were very lowly expressed or exact duplicates (in the protein sequence) of one another. For simplicity, we here show data (GeTMM-normalised) for only the most expressed genes or paralogs with differing expression patterns, but data for all identified globin genes can be found in sheet 1 in S2 Data.

## Statistical analyses

All statistical analyses of the physiological data and the expression of specific globin genes were performed in R (v. 3.6.1; http://www.R-project.org/). LMs were used to determine how physiological metrics changed as a function of age and mass, and the best model was selected based on the lowest Akaike information criterion (AIC) using the MuMIn package [86]. Age acted as a categorical variable when analysing swimming, oxygen uptake, hypoxia tolerance experiments (LOE being the response variable), and gene expression (Hb, Mb, Cytgb, and Ngb expression acted as response variables). The best model fit was validated using Dharma residuals using the Dharma package [87]. Upper and lower confidence intervals, as well as *post hoc* tests determining differences in physiological metrics (*U*_*crit*_, oxygen uptake [SMR, MMR, AS], hypoxia tolerance, and gene expression) when age acted as a categorical variable (α = 0.05), were determined using the emmeans package [88]. Generally, oxygen uptake (e.g., SMR and MMR) was measured in relation to changes in body mass to examine the scaling exponent used to predict how oxygen demands are expected to change as fish develop and grow. However, we predicted *a priori* that mass may likely be variable over age (S1 Fig), and distinct changes in oxygen uptake, hypoxia tolerance, and gene expression of oxygen-carrying proteins will occur at specific ages. We show the mass scaling exponent by plotting mass-specific oxygen uptake rates (mg O_2_ g^−1^ h^−1^) for SMR and MMR against body mass (mg) to predict how oxygen uptake rates are expected to change as fish age (S2 and S3 Figs). We determined the scaling exponent by fitting an exponential curve (y = ax^b^, where y is mass-specific oxygen uptake (mg O_2_ g^−1^ h^−1^), a is the intercept, x is mass (mg), and b is the scaling exponent) to the data. The scaling exponents for anemonefish larvae are different than traditional scaling exponents [57,73]. We modelled changes in mass-specific oxygen uptake rates for SMR, MMR, and AS over age (categorical variable) to determine any potential differences in oxygen uptake rates that may be related to age, which would be used to parametrize our hypoxia experiments and when we should collect samples for gene expression.

## Supporting information

S1 Data Contains data used for Fig 2 (sheet 1: critical swimming speed in BL s^−1^; sheet 2: SMR and MMR; sheet 3: hypoxia tolerance) and Fig 3 (sheet 4: gene expression of Hb, Mb, Cytgb, and Ngb), as well as S1 Fig (sheet 2: age versus mass), S2 Fig (sheet 1: critical swimming speed, cm s^−1^), S3 Fig (sheet 2: SMR versus mass), S4 Fig (sheet 2: MMR versus mass), and S5 Fig (sheet 2: AAS and FAS). AAS, absolute aerobic scope; BL, body length; Cytgb, cytoglobin; FAS, factorial aerobic scope; Hb, haemoglobin; Mb, myoglobin; MMR, maximum metabolic rate; Ngb, neuroglobin; SMR, standard metabolic rate.
(XLSX)

S2 Data Expression and statistical results from transcriptomic analysis presented in Fig 4. Sheet 1. Haemoglobin_getmm. GeTMM-normalised expression and gene IDs for Hb (alpha and beta subunits and paralogs i, ii, and iv), Mb, Cytgb, and Ngb for each individual larval anemonefish at ages 4, 6, and 9 dph. These data are the same as S1 Data sheet “Fig 3” but include Ensembl gene IDs. Sheet 2. Raw_counts. Raw, nonnormalised gene counts for all genes from the FeatureCount pipeline (Fig 4A). Sheet 3. all_degs_getmm. GeTMM-normalised expression and gene IDs (with abbreviations and descriptions) for genes identified as DEGs, for each individual fish larvae across 3 age groups (4, 6, and 9 dph) (Fig 4B). Sheet 4. Heatmap_toptags. GeTMM-normalised expression and gene IDs (with abbreviations and descriptions) for the top most significant genes across 3 age groups (4, 6, and 9 dph), used to create Heatmap (Fig 4D). Sheet 5. Volcanoplot_toptag. Mean across all groups (“baseMean”), log10 of the mean (“log10basemean_amel”), log of the fold-change (“log2FoldChange”), error estimate of the log fold-change (lfcSE), significance (“pvalue”), and adjusted *p*-value (“padj”) for the top most significant DEGs when comparing 9 to 4 dph, used to create Volcano plot (Fig 4E). Sheet 6. GOs. GO enrichment analyses (of the significantly up- or down-regulated genes when comparing 9 dph and 4 dph. Data include the fold enrichment (how many more times than expected did a gene belonging to a given GO category appear in the list of DEGs) and the adjusted *p*-value (FDR) (Fig 4F). Sheet 7a. 6dph_vs_4dph, 7b. 9dph_vs_4dph, 7c. 9dph_vs_6dph. Mean across all groups (“baseMean”), log10 of the mean (“log10basemean_amel”), log of the fold-change (“log2FoldChange”), error estimate of the log fold-change (lfcSE), significance (“pvalue”), and adjusted *p*-value (“padj”) for all DEGs identified when comparing 9 dph and 4 dph, 9 dph and 6 dph, and 6 dph and 4 dph. Sheet 8a. down_unique_6dph_vs_4dph, Sheet 8b. up_unique_6dph_vs_4dph. Mean across all groups (“baseMean”), log10 of the mean (“log10basemean_amel”), log of the fold-change (“log2FoldChange”), error estimate of the log fold-change (lfcSE), significance (“pvalue”), and adjusted *p*-value (“padj”) for DEGs that were unique when comparing 6 dph and 4 dph, i.e., down or up at 6 dph but not 9 dph. Sheet 9a. down_unique_9dph_vs_6dph, Sheet 9b. up_unique_9dph_vs_6dph. Mean across all groups (“baseMean”), log10 of the mean (“log10basemean_amel”), log of the fold-change (“log2FoldChange”), error estimate of the log fold-change (lfcSE), significance (“pvalue”), and adjusted *p*-value (“padj”) for DEGs that were unique when comparing 9 dph and 6 dph, i.e., down or up at 9 dph but not at 6 dph.
(XLSX)

S1 StatisticalOutput List of model summaries and pairwise comparisons for Figs 2A, 2B, 2C, 2D, S3A, 3B, 3C, 3D, 3E, 3F, 3G, 3H, 3I, 3S, S2, S5A, and S5B.
(DOCX)

S1 Metadata Description of metadata for each fish used in the study, and definition of variables.
(DOCX)

S1 Fig Relationship between age (dph) and mass (mg) for the anemonefish (*Amphiprion melanopus*) over the entire larval duration (1–9 dph; *n* = 8–10 individuals per day) during swimming respirometry experiments. LMs are present with 95% confidence intervals. The regression equation is based on the best fitting LM: mass = 0.422(age)+0.0328; r^2^ = 0.65). The data underlying this figure can be found on sheet 2 in S1 Data. dph, days post hatch; LM, linear model.
(TIF)

S2 Fig Relationship between age (dph) and critical swimming speed (cm s^−1^) for the anemonefish (*Amphiprion melanopus*) over the entire larval duration (1–9 dph; *n* = 8–10 individuals per day) during swimming respirometry experiments). Each point is colour coded to represent the size range (length; cm) of the individual larva. Boxplots show median and interquartile ranges, and “X” indicates average age (dph), and different lowercase letters represent statistical differences (LMs; α = 0.05). The data underlying this figure can be found on sheet 1 in S1 Data, and details on statistical output can be found in the supporting information S1 StatisticalOutput. dph, days post hatch; LM, linear model.
(TIF)

S3 Fig Relationship between mass-specific SMR (mg O2 g^−1^ h^−1^) and mass (mg) for the anemonefish (*Amphiprion melanopus*) over the entire larval duration (1–9 dph; *n* = 8–10 individuals per day) during swimming respirometry experiments (see Methods for details). Each point is colour coded to represent the age (dph) of the individual larva. Model predictions are presented with 95% confidence intervals. The equation for the exponential curve fit is present on the figure showing the scaling exponent for how mass-specific SMR is predicted to change with mass (r^2^ = 0.47). The data underlying this figure can be found on sheet 2 in S1 Data. dph, days post hatch; SMR, standard metabolic rate.
(TIF)

S4 Fig Relationship between mass-specific MMR (mg O2 g^−1^ h^−1^) and mass (mg) for the anemonefish (*Amphiprion melanopus*) over the entire larval duration (1–9 dph; *n* = 8–10 individuals per day) during swimming respirometry experiments (see Methods for details). Each point is colour coded to represent the age (dph) of the individual larva. Model predictions are presented with 95% confidence intervals. The equation for the exponential curve fit is present on the figure showing the scaling exponent for how mass-specific MMR is predicted to change with mass (r^2^ = 0.39). The data underlying this figure can be found on sheet 2 in S1 Data. dph, days post hatch; MMR, maximum metabolic rate.
(TIF)

S5 Fig Relationship between (A) AAS (mg O2 g^−1^ h^−1^) and (B) FAS with age (dph) over the entire larval duration of the anemonefish (*Amphiprion melanopus*). Each individual point represents calculations from oxygen uptake rates from each individual larva (*n* = 8–10 per age) swum under a swimming respirometry protocol (see Methods for details) to achieve simultaneous measures of swimming speed and oxygen uptake rates. Each point is colour coded to represent the size range (mass; mg) of the individual larva. Boxplots show median and interquartile ranges, and “X” indicates average AAS or FAS per age (dph). The data underlying this figure can be found on sheet 2 in S1 Data, and details on statistical output can be found in the supporting information S1 StatisticalOutput. AAS, absolute aerobic scope; dph, days post hatch; FAS, factorial aerobic scope.
(TIF)

## Abbreviations

AAS: absolute aerobic scope
AS: aerobic scope
ATP: adenosine triphosphate
BL: body length
Cytgb: cytoglobin
DEG: differentially expressed gene
dph: days post hatch
FAS: factorial aerobic scope
GO: gene ontology
GTP: guanosine triphosphate
Hb: haemoglobin
LM: linear model
LOE: loss of equilibrium
Mb: myoglobin
MMR: maximum metabolic rate
Ngb: neuroglobin
SMR: standard metabolic rate

## References

[1] Rabosky DL, Chang J, Title PO, Cowman PF, Sallan L, Friedman M, et al. An inverse latitudinal gradient in speciation rate for marine fishes. Nature. 2018;559:392–395. doi: 10.1038/s41586-018-0273-1 29973726

[2] Hughes TP, Rodrigues MJ, Bellwood DR, Ceccarelli D, Hoegh-Guldberg O, McCook L, et al. Phase Shifts, Herbivory, and the Resilience of Coral Reefs to Climate Change. Curr Biol. 2007;17:360–365. doi: 10.1016/j.cub.2006.12.049 17291763

[3] Leis JM, McCormick MI. The Biology, Behavior, and Ecology of the Pelagic, Larval Stage of Coral Reef Fishes. Coral Reef Fishes. Elsevier; 2002. p. 171–199. doi: 10.1016/b978-012615185-5/50011-6

[4] Leis JM. Are Larvae of Demersal Fishes Plankton or Nekton? Adv Mar Biol. 2006;51:57–141. doi: 10.1016/S0065-2881(06)51002-8 16905426

[5] Downie AT, Leis JM, Cowman PF, McCormick MI, Rummer JL. The influence of habitat association on swimming performance in marine teleost fish larvae. Fish Fish. 2021;22:1187–1212. doi: 10.1111/faf.12580

[6] Downie AT, Illing B, Faria AM, Rummer JL. Swimming performance of marine fish larvae: review of a universal trait under ecological and environmental pressure. Rev Fish Biol Fish. 2020:93–108. doi: 10.1007/s11160-019-09592-w

[7] Stobutzki IC, Bellwood DR. An analysis of the sustained swimming abilities of pre- and post-settlement coral reef fishes. J Exp Mar Biol Ecol. 1994;175:275–286. doi: 10.1016/0022-0981(94)90031-0

[8] Leis JM, Sweatman HPA, Reader SE. What the pelagic stages of coral reef fishes are doing out in blue water: Daytime field observations of larval behavioural capabilities. Mar Freshw Res. 1996;47:401–411. doi: 10.1071/MF9960401

[9] Nilsson GE, Östlund-Nilsson S, Penfold R, Grutter AS. From record performance to hypoxia tolerance: Respiratory transition in damselfish larvae settling on a coral reef. Proc R Soc B Biol Sci. 2007;274:79–85. doi: 10.1098/rspb.2006.3706 17015334 PMC1679883

[10] Mandic M, Todgham AE, Richards JG. Mechanisms and evolution of hypoxia tolerance in fish. Proc R Soc B Biol Sci. 2009;276:735–744. doi: 10.1098/rspb.2008.1235 18996831 PMC2660936

[11] Nilsson GE, Hobbs JPA, Östlund-Nilsson S. Tribute to P. L. Lutz: Respiratory ecophysiology of coral-reef teleosts. J Exp Biol. 2007;210:1673–1686. doi: 10.1242/jeb.02718 17488931

[12] Nilsson GE, Östlund-Nilsson S. Hypoxia in paradise: Widespread hypoxia tolerance in coral reef fishes. Proc R Soc B Biol Sci. 2004:271. doi: 10.1098/rsbl.2003.0087 15101411 PMC1810002

[13] Xiao W. The hypoxia signalling pathway and hypoxic adaptation in fishes. Science China Life Sciences. Science in China Press; 2015. p. 148–155. doi: 10.1007/s11427-015-4801-z 25595051

[14] Giles MA, Randall DJ. Oxygenation characteristics of the polymorphic hemoglobins of coho salmon (Oncorhynchus kisutch) at different developmental stages. Comp Biochem Physiol A Physiol. 1980;65:265–271. doi: 10.1016/0300-9629(80)90029-8

[15] de Souza PC, Bonilla-Rodriguez GO. Fish hemoglobins. Braz J Med Biol Res. 2007;40:769–778. doi: 10.1590/s0100-879x2007000600004 17581674

[16] Giles MA, Vanstone WE. Ontogenetic Variation in the Multiple Hemoglobins of Coho Salmon (Oncorhynchus kisutch) and Effect of Environmental Factors on Their Expression. J Fish Res Bd Can. 1976;33:1144–1149. doi: 10.1139/f76-143

[17] Helbo S, Dewilde S, Williams DR, Berghmans H, Berenbrink M, Cossins AR, et al. Functional differentiation of myoglobin isoforms in hypoxia-tolerant carp indicates tissue-specific protective roles. Am J Physiol Regul Integr Comp Physiol. 2012;302:693–701. doi: 10.1152/ajpregu.00501.2011 22170621

[18] Keppner A, Maric D, Correia M, Koay TW, Orlando IMC, Vinogradov SN, et al. Lessons from the post-genomic era: Globin diversity beyond oxygen binding and transport. Redox Biology. Elsevier B.V.; 2020. doi: 10.1016/j.redox.2020.101687 32863222 PMC7475203

[19] Zweier JL, Hemann C, Kundu T, Ewees MG, Khaleel SA, Samouilov A, et al. Cytoglobin has potent superoxide dismutase function. Proc Nat Ac Sci. 2021:118. doi: 10.1073/pnas.2105053118 34930834 PMC8719900

[20] Bateman A, Martin M-J, Orchard S, Magrane M, Ahmad S, Alpi E, et al. UniProt: the Universal Protein Knowledgebase in 2023. Nucleic Acids Res. 2023;51:D523–D531. doi: 10.1093/nar/gkac1052 36408920 PMC9825514

[21] Matsuda K, Yuzaki M. Cbln family proteins promote synapse formation by regulating distinct neurexin signalling pathways in various brain regions. Eur J Neurosci. 2011;33:1447–1461. doi: 10.1111/j.1460-9568.2011.07638.x 21410790

[22] Verhoeven K, van Laer L, Kirschhofer K, Legan PK, Hughes DC, Schatteman I, et al. Mutations in the human α-tectorin gene cause autosomal dominant non-syndromic hearing impairment. Nat Genet. 1998;19:60–62. doi: 10.1038/ng0598-60 9590290

[23] Leis JM, Fisher R. Swimming speed of settlement-stage reef-fish larvae measured in the laboratory and in the field: a comparison of critical speed and in situ speed. Proceedings of the 10th International Coral Reef Symposium. 2006;445:438–445.

[24] Jones GP, Millcich MJ, Emsile MJ, Lunow C. Self-recruitment in a coral fish population. Nature. 1999;402:802–804. doi: 10.1038/45538

[25] Jones GP, Planes S, Thorrold SR. Coral reef fish larvae settle close to home. Curr Biol. 2005;15:1314–1318. doi: 10.1016/j.cub.2005.06.061 16051176

[26] Prescott LA, Regish AM, McMahon SJ, McCormick SD, Rummer JL. Rapid embryonic development supports the early onset of gill functions in two coral reef damselfishes. J Exp Biol. 2021:224. doi: 10.1242/jeb.242364 34708857

[27] Roux N, Salis P, Lambert A, Logeux V, Soulat O, Romans P, et al. Staging and normal table of postembryonic development of the clownfish (Amphiprion ocellaris). Dev Dyn. 2019;248:545–568. doi: 10.1002/dvdy.46 31070818 PMC6771578

[28] Oikawa S, Itazawa Y, Gotoh M. Ontogenetic change in the relationship between metabolic rate and body mass in a sea bream Pagrus major (Temminck & Schlegel). J Fish Biol. 1991;38:483–496. doi: 10.1111/j.1095-8649.1991.tb03136.x

[29] Fisher R, Bellwood DR, Job SD. Development of swimming abilities in reef fish larvae. Mar Ecol Prog Ser. 2000;202:163–173.

[30] Illing B, Severati A, Hochen J, Boyd P, Raison P, Mather R, et al. Automated flow control of a multi-lane swimming chamber for small fishes indicates species-specific sensitivity to experimental protocols. Conserv Physiol. 2020;9:1–16. doi: 10.1093/conphys/coaa131 33659062 PMC7905161

[31] White CR, Alton LA, Bywater CL, Lombardi EJ, Marshall DJ. Metabolic scaling is the product of life-history optimization. Science. 2022;377:834–839. doi: 10.1126/science.abm7649 35981018

[32] Mazurais D, Darias M, Zambonino-Infante JL, Cahu CL. Transcriptomics for understanding marine fish larval development. Can J Zool. 2011:599–611. doi: 10.1139/z11-036

[33] Darias MJ, Zambonino-Infante JL, Hugot K, Cahu CL, Mazurais D. Gene expression patterns during the larval development of European sea bass (Dicentrarchus Labrax) by microarray analysis. Mar Biotechnol. 2008;10:416–428. doi: 10.1007/s10126-007-9078-1 18246396

[34] Ferraresso S, Bonaldo A, Parma L, Cinotti S, Massi P, Bargelloni L, et al. Exploring the larval transcriptome of the common sole (Solea solea L.). BMC Genomics. 2013:14. doi: 10.1186/1471-2164-14-315 23663263 PMC3659078

[35] Alves RN, Gomes AS, Stueber K, Tine M, Thorne MAS, Smáradóttir H, et al. The transcriptome of metamorphosing flatfish. BMC Genomics. 2016:17. doi: 10.1186/s12864-016-2699-x 27233904 PMC4884423

[36] Tang X, Jiang S, Wang H, Zhou Y, Peng F, Zhang X, et al. Transcriptome Sequencing Analysis Reveals Dynamic Changes in Major Biological Functions during the Early Development of Clearhead Icefish, Protosalanx chinensis. Fishes. 2022:7. doi: 10.3390/fishes7030115

[37] Janiak MC, Pinto SL, Duytschaever G, Carrigan MA, Melin AD. Genetic evidence of widespread variation in ethanol metabolism among mammals: revisiting the ‘myth” of natural intoxication.’. Biol Lett. 2020;16:20200070. doi: 10.1098/rsbl.2020.0070 32343936 PMC7211468

[38] Eriksson K, Nummi H. Alcohol accumulation from ingested berries and alcohol metabolism in passerine birds. Ornis Fenn. 1982;2–6.

[39] Val A. Organic phosphates in the red blood cells of fish. Comp Biochem Physiol A Mol Integr Physiol. 2000;125:417–435. doi: 10.1016/s1095-6433(00)00184-7 10840217

[40] Rummer JL, McKenzie DJ, Innocenti A, Supuran CT, Brauner CJ. Root Effect Hemoglobin May Have Evolved to Enhance General Tissue Oxygen Delivery. Science. 2013;340:1327–1329. doi: 10.1126/science.1233692 23766325

[41] Val AL, Gomes KRM, de Almeida-Val VMF. Rapid regulation of blood parameters under acute hypoxia in the Amazonian fish Prochilodus nigricans. Comp Biochem Physiol A Mol Integr Physiol. 2015;184:125–131. doi: 10.1016/j.cbpa.2015.02.020 25737030

[42] Mania M, Bruschetta G, Avenoso A, D’Ascola A, Scuruchi M, Campo A, et al. Evidence for embryonic haemoglobins from Sparus aurata under normal and hypoxic conditions. Fish Physiol Biochem. 2019;45:943–954. doi: 10.1007/s10695-018-0605-y 30627834

[43] Vanstone WE, Roberts E, Tsuyuki H. Changes in the multiple hemoglobin patterns of some Pacific salmon. Can J Physiol Pharmacol. 1964;42:697–703. doi: 10.1139/y64-079 14324202

[44] Sullivan CV, Dickhoff WW M, Hersbberger WK. Changes in the hemoglobin system of the coho salmon Oncorhynchus kisutch during smoltification and triiodothyronine and propylthiouracil treatment. Comp Biochem Physiol A Physiol. 1985;81:807–813. doi: 10.1016/0300-9629(85)90911-9 2863070

[45] Tiedke J, Gerlach F, Mitz SA, Hankeln T, Burmester T. Ontogeny of globin expression in zebrafish (Danio rerio). J Comp Physiol B. 2011;181:1011–1021. doi: 10.1007/s00360-011-0588-9 21614507

[46] Brownlie A, Hersey C, Oates AC, Paw BH, Falick AM, Witkowska HE, et al. Characterization of embryonic globin genes of the zebrafish. Dev Biol. 2003;255:48–61. doi: 10.1016/s0012-1606(02)00041-6 12618133

[47] Gallagher MD, Macqueen DJ. Evolution and expression of tissue globins in ray-finned fishes. Genome Biol Evol. 2017;9:32–47. doi: 10.1093/gbe/evw266 28173090 PMC5381549

[48] Helbo S, Weber RE, Fago A. Expression patterns and adaptive functional diversity of vertebrate myoglobins. Biochimica et Biophysica Acta–Proteins and Proteomics. Elsevier B.V.; 2013. p. 1832–1839. doi: 10.1016/j.bbapap.2013.01.037 23388387

[49] Tian R, Wang Z, Niu X, Zhou K, Xu S, Yang G. Evolutionary genetics of hypoxia tolerance in cetaceans during diving. Genome Biol Evol. 2016;8:827–839. doi: 10.1093/gbe/evw037 26912402 PMC4824146

[50] Jaspers RT, Testerink J, Della Gaspera B, Chanoine C, Bagowski CP, van der Laarse WJ. Increased oxidative metabolism and myoglobin expression in zebrafish muscle during chronic hypoxia. Biol Open. 2014;3:718–727. doi: 10.1242/bio.20149167 25063194 PMC4133725

[51] Burmester T, Hankeln T. Neuroglobin: A respiratory protein of the nervous system. News Physiol Sci. 2004:110–113. doi: 10.1152/nips.01513.2003 15143204

[52] Awenius C, Hankeln T, Burmester T. Neuroglobins from the zebrafish Danio rerio and the pufferfish Tetraodon nigroviridis. Biochem Biophys Res Commun. 2001;287:418–421. doi: 10.1006/bbrc.2001.5614 11554744

[53] Hankeln T, Ebner B, Fuchs C, Gerlach F, Haberkamp M, Laufs T, et al. Neuroglobin and cytoglobin in search of their role in the vertebrate globin family. J Inorg Biochem. 2005;99:110–119. doi: 10.1016/j.jinorgbio.2004.11.009 15598495

[54] Peck MA, Moyano M. Measuring respiration rates in marine fish larvae: Challenges and advances. J Fish Biol. 2016;88:173–205. doi: 10.1111/jfb.12810 26768975

[55] McLeod IM, Rummer JL, Clark TD, Jones GP, McCormick MI, Wenger AS, et al. Climate change and the performance of larval coral reef fishes: The interaction between temperature and food availability. Conserv Physiol. 2013;1:1–12. doi: 10.1093/conphys/cot024 27293608 PMC4732438

[56] Wieser W. Energetics of fish larvae, the smallest vertebrates. Acta Physiologica Scandinavica. 1995;154:279–290. doi: 10.1111/j.1748-1716.1995.tb09912.x 7572226

[57] Killen SS, Costa I, Brown JA, Gamperl AK. Little left in the tank: Metabolic scaling in marine teleosts and its implications for aerobic scope. Proc R Soc B: Biol Sci. 2007;274:431–438. doi: 10.1098/rspb.2006.3741 17164208 PMC1702384

[58] Illing B, Downie AT, Beghin M, Rummer JL. Critical thermal maxima of early life stages of three tropical fishes: Effects of rearing temperature and experimental heating rate. J Therm Biol. 2020;90:102582. doi: 10.1016/j.jtherbio.2020.102582 32479385

[59] Rummer JL, Couturier CS, Stecyk JAW, Gardiner NM, Kinch JP, Nilsson GE, et al. Life on the edge: thermal optima for aerobic scope of equatorial reef fishes are close to current day temperatures. Glob Chang Biol. 2014;20:1055–1066. doi: 10.1111/gcb.12455 24281840 PMC4677772

[60] Nilsson GE, Crawley N, Lunde IG, Munday PL. Elevated temperature reduces the respiratory scope of coral reef fishes. Glob Chang Biol. 2009;15:1405–1412. doi: 10.1111/j.1365-2486.2008.01767.x

[61] Hess S, Prescott LJ, Hoey AS, McMahon SA, Wenger AS, Rummer JL. Species-specific impacts of suspended sediments on gill structure and function in coral reef fishes. Proc R Soc B Biol Sci. 2017;284:20171279. doi: 10.1098/rspb.2017.1279 29093217 PMC5698636

[62] Fisher R, Bellwood D. Effects of feeding on the sustained swimming abilities of late-stage larval Amphiprion melanopus. Coral Reefs. 2001;20:151–154. doi: 10.1007/s003380100149

[63] Fisher R, Leis JM, Clark DL, Wilson SK. Critical swimming speeds of late-stage coral reef fish larvae: Variation within species, among species and between locations. Mar Biol. 2005;147:1201–1212. doi: 10.1007/s00227-005-0001-x

[64] Almany GR, Planes S, Thorrold SR, Berumen ML, Bode M, Saenz-Agudelo P, et al. Larval fish dispersal in a coral-reef seascape. Nat Ecol Evol. 2017;1:1–7. doi: 10.1038/s41559-017-0148 28812625

[65] Planes S, Jones GP, Thorrold SR. Larval dispersal connects fish populations in a network of marine protected areas. Proc Natl Acad Sci. 2009;106:5693–5697. doi: 10.1073/pnas.0808007106 19307588 PMC2659712

[66] Saenz-Agudelo P, Jones GP, Thorrold SR, Planes S. Connectivity dominates larval replenishment in a coastal reef fish metapopulation. Proc R Soc B Biol Sci. 2011;278:2954–2961. doi: 10.1098/rspb.2010.2780 21325328 PMC3151711

[67] Almany GR, Berumen ML, Thorrold SR, Planes S, Jones GP. Local replenishment of coral reef fish populations in a marine reserve. Science. 2007;316:742–744. doi: 10.1126/science.1140597 17478720

[68] Berumen ML, Almany GR, Planes S, Jones GP, Saenz-Agudelo P, Thorrold SR. Persistence of self-recruitment and patterns of larval connectivity in a marine protected area network. Ecol Evol. 2012;2:444–452. doi: 10.1002/ece3.208 22423335 PMC3298954

[69] Rummer JL, Binning SA, Roche DG, Johansen JL. Methods matter: Considering locomotory mode and respirometry technique when estimating metabolic rates of fishes. Conserv Physiol. 2016;4:1–13. doi: 10.1093/conphys/cow008 27382471 PMC4922262

[70] Brett JR. The Respiratory Metabolism and Swimming Performance of Young Sockeye Salmon. J Fish Res Bd Can. 1964;21:1183–1226. doi: 10.1139/f64-103

[71] Downie AT, Kieffer JD. Swimming performance in juvenile shortnose sturgeon (Acipenser brevirostrum): The influence of time interval and velocity increments on critical swimming tests. Conserv Physiol. 2017:5. doi: 10.1093/conphys/cox038 28835841 PMC5550615

[72] Hunt Von Herbing I, Boutilier RG. Activity and metabolism of larval Atlantic cod (Gadus morhua) from Scotian Shelf and Newfoundland source populations. Mar Biol. 1996:607–617. doi: 10.1007/bf00351042

[73] Post JR, Lee A. Metabolic ontogeny of teleost fishes. Can J Fish Aqua Sci. 1996;53:910–923. doi: 10.1139/f95-278

[74] Andrews S. FastQC: a quality control tool for high throughput sequence data. 2010.

[75] Dobin A, Davis CA, Schlesinger F, Drenkow J, Zaleski C, Jha S, et al. STAR: Ultrafast universal RNA-seq aligner. Bioinformatics. 2013;29:15–21. doi: 10.1093/bioinformatics/bts635 23104886 PMC3530905

[76] Cunningham F, Allen JE, Allen J, Alvarez-Jarreta J, Amode MR, Armean IM, et al. Ensembl 2022. Nucleic Acids Res. 2022;50:D988–D995. doi: 10.1093/nar/gkab1049 34791404 PMC8728283

[77] Liao Y, Smyth GK, Shi W. FeatureCounts: An efficient general purpose program for assigning sequence reads to genomic features. Bioinformatics. 2014;30:923–930. doi: 10.1093/bioinformatics/btt656 24227677

[78] Smid M, Coebergh van den Braak RRJ, van de Werken HJG, van Riet J, van Galen A, de Weerd V, et al. Gene length corrected trimmed mean of M-values (GeTMM) processing of RNA-seq data performs similarly in intersample analyses while improving intrasample comparisons. BMC Bioinformatics. 2018:19. doi: 10.1186/s12859-018-2246-7 10.1186/s12859-018-2246-7 PMC6013957 29929481

[79] McCarthy DJ, Chen Y, Smyth GK. Differential expression analysis of multifactor RNA-Seq experiments with respect to biological variation. Nucleic Acids Res. 2012;40:4288–4297. doi: 10.1093/nar/gks042 22287627 PMC3378882

[80] Love MI, Huber W, Anders S. Moderated estimation of fold change and dispersion for RNA-seq data with DESeq2. Genome Biol. 2014:15. doi: 10.1186/s13059-014-0550-8 25516281 PMC4302049

[81] Yu G, Lam TTY, Zhu H, Guan Y. Two methods for mapping and visualizing associated data on phylogeny using GGTree. Mol Biol Evol. 2018;35:3041–3043. doi: 10.1093/molbev/msy194 30351396 PMC6278858

[82] Chen H, Boutros PC. VennDiagram: A package for the generation of highly-customizable Venn and Euler diagrams in R. BMC Bioinformatics. 2011:12. doi: 10.1186/1471-2105-12-35 21269502 PMC3041657

[83] Gu Z, Eils R, Schlesner M. Complex heatmaps reveal patterns and correlations in multidimensional genomic data. Bioinformatics. 2016;32:2847–2849. doi: 10.1093/bioinformatics/btw313 27207943

[84] Young MD, Wakefield MJ, Smyth GK, Oshlack A. Open Access METHOD Gene ontology analysis for RNA-seq: accounting for selection bias Goseq Goseq is a method for GO analysis of RNA-seq data that takes into account the length bias inherent in RNA-seq. Genome Biol. 2010. Available: http://genomebiology.com/2010/11/2/R14. 10.1186/gb-2010-11-2-r14 PMC2872874 20132535

[85] Durinck S, Moreau Y, Kasprzyk A, Davis S, de Moor B, Brazma A, et al. BioMart and Bioconductor: A powerful link between biological databases and microarray data analysis. Bioinformatics. 2005;21:3439–3440. doi: 10.1093/bioinformatics/bti525 16082012

[86] Barton, K. MuMIn: multi-model inference. R package version 1. 0. 0. http://r-forge.r-project.org/projects/mumin/. 2009 [cited 2020 Jul 17]. Available from: https://ci.nii.ac.jp/naid/10030918982

[87] Hartig F, Hartig MF. Package “DHARMa.” Vienna, Austria: R Development Core Team; 2017.

[88] Lenth RV. Least-squares means: The R package lsmeans. J Stat Softw. 2016:69. doi: 10.18637/jss.v069.i01

