# Supplementary material for: Rapid physiological and transcriptomic changes associated with oxygen delivery in larval anemonefish suggest a role in adaptation to life on hypoxic coral reefs
Source: PLoS Biol. 2023 May 11;21(5):e3002102. doi: 10.1371/journal.pbio.3002102 (PMC10174562; doi:10.1371/journal.pbio.3002102)
Supplement: S1 StatisticalOutput — List of model summaries and pairwise comparisons for Figs 2A, 2B, 2C, 2D, S3A, 3B, 3C, 3D, 3E, 3F, 3G, 3H, 3I, 3S, S2, S5A, and S5B. (DOCX) [file pbio.3002102.s003.docx]

**Rapid physiological and transcriptomic changes associated with oxygen delivery in larval anemonefish suggest a role in adaptation to life on hypoxic coral reefs**

Adam T. Downie^1,2*^, Sjannie Lefevre^3^, Björn Illing^1,4^, Jessica Harris^1^, Michael D. Jarrold^1,5^, Mark I. McCormick^6^, Göran E. Nilsson^3^, Jodie L. Rummer^1.5^

^1^Australian Research Council Centre of Excellence for Coral Reef Studies, James Cook University, Townsville, QLD, Australia

^2^School of Biological Sciences, University of Queensland, St. Lucia, QLD, Australia

^3^Section for Physiology and Cell Biology, Department of Biosciences, University of Oslo, Oslo, Norway

^4^Thünen Institute of Fisheries Ecology, Bremerhaven, Germany

^5^College of Science and Engineering, James Cook University, Townsville, QLD, Australia

^6^Coastal Marine Field Station, School of Science, University of Waikato, Tauranga, New Zealand

^*^corresponding author: adam.downie@uq.edu.au

**Abstract**

Connectivity of coral reef fish populations relies on successful dispersal of a pelagic larval phase. Pelagic larvae must exhibit high swimming abilities to overcome ocean and reef currents, but once settling onto the reef, larvae transition to endure habitats that become hypoxic at night. Therefore, coral reef fish larvae must rapidly and dramatically shift their physiology over a short period of time. Taking an integrative physiological approach, using swimming respirometry and examining hypoxia tolerance and transcriptomics, we show that reef fish larvae rapidly transition between ‘physiological extremes’ at the end of their larval phase. Daily measurements of swimming larval anemonefish over their entire early development show that they initially have very high mass-specific oxygen uptake rates. However, oxygen uptake rates decrease midway through the larval phase. This occurs in conjunction with a switch in haemoglobin gene expression and increased expression of myoglobin, cytoglobin, and neuroglobin, which may all contribute to the observed increase in hypoxia tolerance. Our findings indicate that critical ontogenetic changes in the gene expression of oxygen-binding proteins may underpin the physiological mechanisms needed for successful larval recruitment to reefs, which in turn supports population growth and connectivity.

**Table of Contents**

Table S1.1 Model Summary (*U*_crit_; BL s^-1^)………………………………………...................3

Table S1.2 Emmeans pairwise comparisons (*U_crit_*; BL s^-1^)……………………......................4

Table S1.3 Model Summary (*U_crit_*; cm s^-1^)…………...………………..……………….…....5

Table S1.4 Model Summary (*U_crit_*; cm s^-1^)………...……………………………...................6

Table S2.1 Model Summary (SMR)………………………………………………….…..….7

Table S2.2 Emmeans pairwise comparisons (SMR)……………………………....................8

Table S3.1 Model Summary (MMR)……………………………………………….…..……9

Table S3.2 Emmeans pairwise comparisons (MMR)……………………………………......9

Table S4.1 Model Summary (Hypoxia)……………………………………………….……10

Table S4.2 Emmeans pairwise comparisons (Hypoxia)…………………………………....10

Table S5.1. Model Summary (hba-i)……………………………………………………….11

Table S5.2 Model Summary (hba-iii)……………………………………………………....11

Table S5.3 Model Summary (hba-iv)………………………………………………………11

Table S5.4 Model Summary (hbb-i)……………………………………………………......11

Table S5.5 Model Summary (hbb-ii)…………………………………………………....….12

Table S5.6 Model Summary (hbb-iv)…………………………………………………..…..12

Table S5.7 Model Summary (ngb)……………………………………………………….....12

Table S5.8 Model Summary (cytgb)………………………………………………..………12

Table S5.9 Model Summary (mb)……………………………………………………….....13

Table S5.10 Emmeans pairwise comparisons (all genes)…………………………………..14

Table S6.1 Model Summary (AAS)…………………………………………………..…….15

Table S6.2 Emmeans pairwise comparisons (AAS)……………………………………......15

Table S7.1 Model Summary (FAS)………………………………………..………….……16

Table S7.2 Emmeans pairwise comparisons (FAS)……………………………………..….16

**Statistical Outputs**

**Table S1.1** Summary statistics of the generalized linear model (glm(log(Ucrit_BL)~age) describing the relationship between the change in critical swimming speed (Ucrit; BL s-1) with age (days post hatch; dph) over the entire larval duration of the anemonefish (*Amphiprion melanopus*)

|  | Estimate | SE | t.value | p-value |
| --- | --- | --- | --- | --- |
| (Intercept) | 0.9 | 0.053 | 17.05 | <0.0001 |
| dph1 | 0.24 | 0.075 | 3.3 | 0.00158 |
| dph2 | 0.4 | 0.075 | 5.3 | <0.0001 |
| dph3 | 0.33 | 0.075 | 4.37 | <0.0001 |
| dph4 | 0.55 | 0.075 | 7.31 | <0.0001 |
| dph5 | 0.44 | 0.075 | 5.95 | <0.0001 |
| dph6 | 0.44 | 0.075 | 5.94 | <0.0001 |
| dph7 | 0.64 | 0.075 | 8.58 | <0.0001 |
| dph8 | 0.62 | 0.075 | 8.21 | <0.0001 |
| dph9 | 1.32 | 0.075 | 17.58 | <0.0001 |

**Table S1.2** Emmeans pairwise comparison, using Tukey’s post-hoc test, of the differences between critical swimming speed (*U_crit_*) in BL s^-1^ and age (dph) over the entire larval duration of the anemonefish (*Amphiprion melanopus*)

| Comparisons | Estimate | SE | df | z-ratio | p-value |
| --- | --- | --- | --- | --- | --- |
| 0dph-1dph | -0.34 | 0.075 | Inf | -5.4 | <0.0001 |
| 0dph-2dph | -0.4 | 0.075 | Inf | -5.3 | <0.0001 |
| 0dph-3dph | -0.33 | 0.075 | Inf | -4.37 | 0.0005 |
| 0dph-4dph | -0.55 | 0.075 | Inf | -7.31 | <0.0001 |
| 0doh-5dph | -0.45 | 0.075 | Inf | -5.95 | <0.0001 |
| 0dph-6dph | -0.44 | 0.075 | Inf | -5.94 | <0.0001 |
| 0dph-7dph | -0.64 | 0.075 | Inf | -8.58 | <0.0001 |
| 0dph-8dph | -0.62 | 0.075 | Inf | -8.21 | <0.0001 |
| 0dph-9dph | -1.32 | 0.075 | Inf | -17.58 | <0.0001 |
| 1dph-2dph | -0.15 | 0.075 | Inf | -2.04 | 0.56 |
| 1dph-3dph | -0.083 | 0.075 | Inf | -1.11 | 0.98 |
| 1dph-4dph | -0.3 | 0.075 | Inf | -4.05 | 0.002 |
| 1dph-5dph | -0.2 | 0.075 | Inf | -2.69 | 0.18 |
| 1dph-6dph | -0.2 | 0.075 | Inf | -2.68 | 0.18 |
| 1dph-7dph | -0.4 | 0.075 | Inf | -5.32 | <0.0001 |
| 1dph-8dph | -0.37 | 0.075 | Inf | -4.96 | <0.0001 |
| 1dph-9dph | -1.07 | 0.075 | Inf | -14.32 | <0.0001 |
| 2dph-3dph | 0.07 | 0.075 | Inf | 0.93 | 0.99 |
| 2dph-4dph | -0.15 | 0.075 | Inf | -2.01 | 0.59 |
| 2dph-5dph | -0.48 | 0.075 | Inf | -0.64 | 0.99 |
| 2dph-6dph | -0.048 | 0.075 | Inf | -0.64 | 0.99 |
| 2dph-7dph | -0.25 | 0.075 | Inf | -3.27 | 0.036 |
| 2dph-8dph | -0.22 | 0.075 | Inf | -2.91 | 0.1 |
| 2dph-9dph | -0.92 | 0.075 | Inf | -12.28 | <0.001 |
| 3dph-4dph | -0.22 | 0.075 | Inf | -2.94 | 0.095 |
| 3dph-5dph | -0.12 | 0.075 | Inf | -1.57 | 0.86 |
| 3dph-6dph | -0.12 | 0.075 | Inf | -1.57 | 0.87 |
| 3dph-7dph | -0.31 | 0.075 | Inf | -4.2 | 0.0011 |
| 3dph-8dph | -0.29 | 0.075 | Inf | -3.84 | 0.0048 |
| 3dph-9dph | -0.99 | 0.075 | Inf | -13.21 | <0.0001 |
| 4dph-5dph | 0.1 | 0.075 | Inf | 1.37 | 0.94 |
| 4dph-6dph | 0.1 | 0.075 | Inf | 1.38 | 0.94 |
| 4dph-7dph | -0.095 | 0.075 | Inf | -1.26 | 0.96 |
| 4dph-8dph | -0.067 | 0.075 | Inf | -0.9 | 0.99 |
| 4dph-9dph | -0.77 | 0.075 | Inf | -10.27 | <0.0001 |
| 5dph-6dph | 0.00049 | 0.075 | Inf | 0.007 | 1.0 |
| 5dph-7dph | -0.2 | 0.075 | Inf | -2.63 | 0.2 |
| 5dph-8dph | -0.17 | 0.075 | Inf | -2.27 | 0.41 |
| 5dph-9dph | -0.87 | 0.075 | Inf | -11.63 | <0.0001 |
| 6dph-7dph | -0.2 | 0.075 | Inf | -2.64 | 0.2 |
| 6dph-8dph | -0.17 | 0.075 | Inf | -2.28 | 0.41 |
| 6dph-9dph | -0.87 | 0.075 | Inf | -11.64 | <0.0001 |
| 7dph-8dph | 0.027 | 0.075 | Inf | 0.36 | 1.0 |
| 7dph-9dph | -0.67 | 0.075 | Inf | -9.0 | <0.0001 |
| 8dph-9dph | -0.7 | 0.075 | Inf | -9.37 | <0.0001 |

**Table S1.3** Summary statistics of the generalized linear model (glm(log(Ucrit)~age) describing the relationship between the change in critical swimming speed (*U*_crit_; cm s^-1^) with age (days post hatch; dph) over the entire larval period of the anemonefish (*Amphiprion melanopus*).

|  | Estimate | SE | t.value | p-value |
| --- | --- | --- | --- | --- |
| (Intercept) | 0.044 | 0.043 | 1.038 | 0.302 |
| dph | 0.168 | 0.0079 | 19.89 | <0.0001 |

**Table S1.4** Emmeans pairwise comparison, using Tukey’s post-hoc test, of the differences between critical swimming speed (*U_crit_*) in cm s^-1^ and age (dph) over the entire larval duration of the anemonefish (*Amphiprion melanopus*)

| Combination | Diff | lwr | upr | p-value |
| --- | --- | --- | --- | --- |
| 1dph - 0dph | 0.373 | -0.568 | 1.31 | 0.954 |
| 2dph - 0dph | 0.465 | -0.476 | 1.41 | 0.843 |
| 3dph – 0dph | 0.54 | -0.401 | 1.48 | 0.695 |
| 4dph – 0dph | 1.03 | 0.0889 | 1.97 | 0.0206 |
| 5dph – 0dph | 1.13 | 0.193 | 2.08 | 0.00657 |
| 6dph – 0dph | 1.21 | 0.273 | 2.15 | 0.00253 |
| 7dph – 0dph | 1.9 | 0.95 | 2.84 | <0.0001 |
| 8dph – 0dph | 2.1 | 1.18 | 3.016 | <0.0001 |
| 9dph – 0dph | 5.58 | 4.64 | 6.52 | <0.0001 |
| 2dph – 1dph | 0.0919 | -0.85 | 1.033 | 0.999 |
| 3dph – 1dph | 0.166 | -0.775 | 1.11 | 0.999 |
| 4dph – 1dph | 0.657 | -0.285 | 1.6 | 0.422 |
| 5dph – 1dph | 0.76 | -0.181 | 1.7 | 0.223 |
| 6dph – 1 dph | 0.841 | -0.1002 | 1.78 | 0.121 |
| 7dph – 1dph | 1.52 | 0.58 | 1.46 | <0.0001 |
| 8dph – 1dph | 1.72 | 0.803 | 2.64 | <0.0001 |
| 9dph – 1dph | 5.21 | 4.27 | 6.15 | <0.0001 |
| 3dph – 2dph | 0.0745 | -0.867 | 1.016 | 0.999 |
| 4dph – 2dph | 0.565 | -0.376 | 1.51 | 0.637 |
| 5dph – 2dph | 0.669 | -0.273 | 1.61 | 0.396 |
| 6dph – 2dph | 0.749 | -0.192 | 1.69 | 0.241 |
| 7dph – 2dph | 1.43 | 0.489 | 2.37 | 0.000156 |
| 8dph – 2dph | 1.63 | 0.71 | 2.55 | <0.0001 |
| 9dph – 2dph | 5.12 | 4.18 | 6.06 | <0.0001 |
| 4dph – 3dph | 0.49 | -0.451 | 1.43 | 0.798 |
| 5dph – 3dph | 0.594 | -0.347 | 1.53 | 0.568 |
| 6dph – 3dph | 0.675 | -0.267 | 1.61 | 0.384 |
| 7dph – 3dph | 1.36 | 0.414 | 2.3 | 0.000424 |
| 8dph – 3dph | 1.56 | 0.636 | 2.48 | 0.0000158 |
| 9dph – 3dph | 5.04 | 4.1 | 5.98 | <0.00001 |
| 5dph – 4dph | 0.104 | -0.837 | 1.05 | 0.999 |
| 6dph – 4dph | 0.184 | -0.757 | 1.13 | 0.999 |
| 7dph – 4dph | 0.865 | -0.0762 | 1.806 | 0.0993 |
| 8dph – 4dph | 1.07 | 0.146 | 1.99 | 0.0107 |
| 9dph - 4dph | 4.55 | 3.61 | 5.5 | <0.00001 |
| 6dph – 5dph | 0.0805 | -0.861 | 1.02 | 0.999 |
| 7dph – 5dph | 0.761 | -0.18 | 1.7 | 0.222 |
| 8dph – 5dph | 0.961 | 0.0419 | 1.88 | 0.0329 |
| 9dph – 5dph | 4.45 | 3.51 | 5.39 | <0.00001 |
| 7dph – 6dph | 0.681 | -0.261 | 1.62 | 0.371 |
| 8dph – 6dph | 0.881 | -0.0386 | 1.8 | 0.072 |
| 9dph – 6dph | 4.37 | 3.42 | 5.31 | <0.0001 |
| 8dph – 7dph | 0.2 | -0.719 | 1.12 | 0.999 |
| 9dph – 7dph | 3.69 | 2.74 | 4.63 | <0.0001 |
| 9dph – 8dph | 3.49 | 2.57 | 4.41 | <0.0001 |

**Table S2.1** Model summary of the linear model (SMR~dph) describing the change in standard metabolic rate (SMR; mg O_2_ g^-1^ h^-1^) with age (days post hatch; dph) over the entire larval duration of the anemonefish (*Amphiprion melanopus*).

|  | Estimate | SE | t.value | p-value |
| --- | --- | --- | --- | --- |
| (Intercept) | 43.05 | 3.94 | 10.936 | <0.0001 |
| dph2 | 4.89 | 5.41 | 0.904 | 0.369 |
| dph3 | -9.16 | 5.41 | -1.69 | 0.0951 |
| dph4 | -3.91 | 5.57 | -0.70 | 0.485 |
| dph5 | -16.7 | 5.41 | -3.09 | 0.00292 |
| dph6 | -28.17 | 5.57 | -5.06 | <0.0001 |
| dph7 | -27.83 | 5.57 | -4.99 | <0.0001 |
| dph8 | -29.73 | 5.28 | -5.63 | <0.0001 |
| dph9 | -30.26 | 5.57 | -5.44 | <0.0001 |

F-statistic: 13.49 on 8 and 68 DF. R^2^=0.61

**Table S2.2.** Emmeans pairwise comparisons of Standard Metabolic Rate (SMR; mg O_2_ g^-1^ h^-1^) with age (days post-hatch; dph) over the entire larval duration of the anemonefish (*Amphiprion melanopus*)

| Comparisons | Diff | Lwr | upr | p-value |
| --- | --- | --- | --- | --- |
| 2dph – 1dph | 4891.9 | -12439.9 | 22223.7 | 0.992 |
| 3dph – 1dph | -9157.6 | -26489.4 | 8174.3 | 0.749 |
| 4dph – 1dph | -3912 | -21746 | 13922.3 | 0.999 |
| 5dph – 1dph | -16703.3 | -34035 | 628.5 | 0.0676 |
| 6dph – 1dph | -28169 | -46004 | -10335.5 | 0.000114 |
| 7dph – 1dph | -27825.4 | -45659.7 | -9991.1 | 0.000143 |
| 9dph – 1dph | -30263.1 | -48097.4 | -12428.8 | 0.00000272 |
| 3dph – 2dph | -14049 | -30863.8 | 2764.9 | 0.176 |
| 4dph – 2dph | -8803.9 | -26135.7 | 8527.9 | 0.786 |
| 5dph – 2dph | -21595.2 | -38409.5 | -4780.9 | 0.00324 |
| 6dph – 2dph | -33061.6 | -50393.4 | -15729.8 | 0.0000019 |
| 7dph – 2dph | -32717.3 | -50049.1 | -15385.5 | 0.0000024 |
| 8dph – 2dph | -34621.5 | -51010.1 | -18232.9 | 0.0000001 |
| 9dph – 2dph | -35155 | -52486.8 | -17823.2 | 0.0000004 |
| 4dph – 3dph | 5245.6 | -12086.3 | 22577.4 | 0.987 |
| 5dph – 3dph | -7545.8 | -24360.1 | 9268.5 | 0.879 |
| 6dph – 3dph | -19012.2 | -36344 | -1680.4 | 0.021 |
| 7dph – 3dph | -18667.8 | -35999.6 | -1336 | 0.025 |
| 8dph – 3dph | -20572 | -36960.6 | -4183.5 | 0.0044 |
| 9dph – 3dph | -21105.6 | -38437.4 | -3773.8 | 0.00649 |
| 5dph – 4dph | -12791.3 | -30123.1 | 4540.5 | 0.32 |
| 6dph – 4dph | -24257.8 | -42092 | -6423.5 | 0.00143 |
| 7dph – 4dph | -23913.4 | -41747.7 | -6079.1 | 0.00177 |
| 8dph – 4dph | -25817.6 | -42736.7 | -8898.5 | 0.000215 |
| 9dph – 4dph | -26351.1 | -44185 | -8516.8 | 0.000378 |
| 6dph – 5dph | -11466.4 | -28798.2 | 5865.4 | 0.469 |
| 7dph – 5dph | -11122 | -28453.9 | 6209.8 | 0.511 |
| 8dph – 5dph | -13026.3 | -29414.8 | 3362.3 | 0.229 |
| 9dph – 5dph | -13559.8 | -30891.6 | 3772 | 0.247 |
| 7dph – 6dph | 344.375 | -17489.9 | 18178.7 | 1.0 |
| 8dph – 6dph | -1559.9 | -18478.9 | 15359.2 | 0.999 |
| 9dph – 6dph | -2093.4 | -19927.7 | 15740.9 | 0.999 |
| 8dph – 7dph | -1904.2 | -18823.3 | 15014.9 | 0.999 |
| 9dph – 7dph | -2437.8 | -20272 | 15396.5 | 0.999 |
| 9dph – 8dph | -533.5 | -17452.6 | 16385.6 | 1.0 |

**Table S3.1** Model summary of the linear model (MMR~dph) describing the change in maximum metabolic rate (MMR; mg O_2_ g^-1^ h^-1^) with age (days post hatch; dph) over the entire larval duration of the anemonefish (*Amphiprion melanopus*).

|  | Estimate | SE | t.value | p-value |
| --- | --- | --- | --- | --- |
| (Intercept) | 72.11 | 5.95 | 12.12 | <0.0001 |
| dph2 | 6.36 | 8.18 | 0.778 | 0.439 |
| dph3 | -8.15 | 8.18 | -0.997 | 0.322 |
| dph4 | -3.35 | 8.41 | -0.399 | 0.691 |
| dph5 | -19.09 | 8.18 | -2.34 | 0.023 |
| dph6 | -48.01 | 8.41 | -5.71 | <0.0001 |
| dph7 | -38.49 | 8.41 | -4.58 | <0.0001 |
| dph8 | -42.27 | 7.98 | -5.3 | <0.0001 |
| dph9 | -39.76 | 8.41 | -4.73 | <0.0001 |

F-Statistic: 13.19 on 8 and 68 DF; R^2^=0.6

**Table S3.2.** Emmeans pairwise comparisons of maximum metabolic rate (MMR; mg O_2_ kg^-1^ h^-1^) with age (days post-hatch; dph) over the entire larval duration of the anemonefish (*Amphiprion melanopus*)

| Comparisons | Diff | lwr | Upr | p-value |
| --- | --- | --- | --- | --- |
| 2dph – 1dph | 6359 | -19833.3 | 32551.3 | 0.997 |
| 3dph – 1dph | -8149.2 | -34341.5 | 18043.1 | 0.985 |
| 4dph – 1dph | -3352.4 | -30304 | 23599.2 | 0.999 |
| 5dph – 1dph | -19091 | -45283.3 | 7101.3 | 0.336 |
| 6dph – 1dph | -48005 | -74956.7 | -21053.4 | 0.0000095 |
| 7dph – 1ph | -38490.2 | -65441.8 | -11538.6 | 0.0000667 |
| 8dph – 1dph | -42266.4 | -67835 | -16697.9 | 0.0000466 |
| 9dph – 1dph | -39755.6 | -66707.2 | -12803.9 | 0.000389 |
| 3dph – 2dph | -14508.2 | -39918.4 | 10902.1 | 0.663 |
| 4dph – 2dph | -9711.4 | -35903.7 | 16480.9 | 0.956 |
| 5dph – 2dph | -25449.9 | -50860.2 | -39.7 | 0.0493 |
| 6dh – 2dph | -54364 | -80556.3 | -28171.7 | 0.0000002 |
| 7dph – 2dph | -44849.2 | -71041.5 | -18656.9 | 0.0000225 |
| 8dph – 2dph | -48625.4 | -73392.3 | -23858.6 | 0.000009 |
| 9dph – 2dph | -46114.5 | -72306.8 | -19922.3 | 0.0000123 |
| 4dph – 3dph | 4796.8 | -21395.5 | 30989.1 | 0.999 |
| 5dph – 3dph | -10941.8 | -36352.1 | 14468.4 | 0.901 |
| 6dph – 3dph | -39855.8 | -66048.1 | -13663.6 | 0.000226 |
| 7dph – 3dph | -30341 | -56533.3 | -4148.7 | 0.0117 |
| 8dph – 3dph | -34117.2 | -58884.1 | -9350.4 | 0.00118 |
| 9dph – 3dph | -31606.4 | -57798.7 | -5414.1 | 0.00725 |
| 5dph – 4dph | -15738.6 | -41930.9 | 10453.7 | 0.599 |
| 6dph – 4dph | -44652.6 | -71604.3 | -17700.9 | 0.0000445 |
| 7dph – 4dph | -35137.8 | -62089.5 | -8186.2 | 0.00264 |
| 8dph – 4dph | -38914 | -64482.6 | -13345.5 | 0.000225 |
| 9dph – 4dph | -36403.2 | -63354.8 | -9451.5 | 0.00158 |
| 6dph – 5dph | -28914 | -55106.3 | -2721.7 | 0.0198 |
| 7dph – 5dph | -19399.2 | -45591.5 | 6793.1 | 0.315 |
| 8dph – 5dph | -23175.4 | -47942.3 | 1591.4 | 0.0845 |
| 9dph – 5dph | -20664.6 | -46856.9 | 5527.7 | 0.238 |
| 7dph – 6dph | 9514.8 | -17436.8 | 36466.5 | 0.967 |
| 8dph – 6dph | 5738.6 | -19829.9 | 31307.2 | 0.998 |
| 9dph – 6dph | 8249.5 | -18702.2 | 35201.1 | 0.986 |
| 8dph – 7dph | -3776.2 | -29344.8 | 21792.3 | 0.999 |
| 9dph – 7dph | -1265.4 | -28217 | 25686.3 | 1.0 |
| 9dph – 8dph | 2510.9 | -23057.7 | 28079.4 | 0.999 |

**Table S4.1** Summary statistics of the linear model (LOE~age) describing the relationship between the air saturation (%) resulting in loss of equilibrium (LOE) of anemonefish (*Amphiprion melanopus*) larvae at ages 4, 6, and 9 days post-hatch (dph) during a hypoxia challenge.

|  | Estimate | SE | t.value | p-value |
| --- | --- | --- | --- | --- |
| (Intercept) | 41.93 | 1.51 | 27.74 | <0.0001 |
| Age6 | -11.24 | 2.18 | -5.15 | <0.0001 |
| Age9 | -18.54 | 2.36 | -7.85 | <0.0001 |

F-Statistic: 32.53 on 2 and 31 DF, r^2^=0.66

**Table S4.2** Emmeans pairwise comparisons from the linear model (LOE~age) comparing the air saturation (%) resulting in loss of equilibrium (LOE) of anemonefish (*Amphiprion melanopus*) larvae at ages 4, 6, and 9 days post-hatch (dph) during a hypoxia challenge.

| Comparison (dph) | Estimate | SE | df | t.ratio | p-value |
| --- | --- | --- | --- | --- | --- |
| 4 – 6 | 11.24 | 2.18 | 31 | 5.15 | <0.0001 |
| 4 – 9 | 18.54 | 2.36 | 31 | 7.85 | <0.0001 |
| 6 – 9 | 7.31 | 2.4 | 31 | 3.04 | 0.0129 |

**Table S5.1** Summary statistics of the linear model (Gene~age) describing the relationship of the expression of the *hba-i* gene from anemonefish (*Amphiprion melanopus*) larvae at ages 4, 6, and 9 days post-hatch (dph).

| Comparison (dph) | Estimate | SE | t.value | p-value |
| --- | --- | --- | --- | --- |
| (Intercept) | 35.02 | 9.65 | 3.63 | 0.0025 |
| Age6 | 17.45 | 13.65 | 1.28 | 0.22 |
| Age9 | 22.97 | 13.65 | 1.68 | 0.11 |

F-statistic: 1.54 on 2 and 15 DF. R^2^ = 0.17

**Table S5.2** Summary statistics of the linear model (Gene~age) describing the relationship of the expression of the *hba-iii* gene from anemonefish (*Amphiprion melanopus*) larvae at ages 4, 6, and 9 days post-hatch (dph).

| Comparison (dph) | estimate | SE | t.value | p-value |
| --- | --- | --- | --- | --- |
| (Intercept) | 36.46 | 6.56 | 5.55 | <0.0001 |
| Age6 | 19.56 | 9.28 | 2.11 | 0.052 |
| Age9 | -14.78 | 9.28 | -1.59 | 0.13 |

F-statistic: 6.89 on 2 and 15 DF, R^2^ = 0.48

**Table S5.3** Summary statistics of the linear model (Gene~age) describing the relationship of the expression of the *hba-iv* gene from anemonefish (*Amphiprion melanopus*) larvae at ages 4, 6, and 9 days post-hatch (dph).

| Comparison (dph) | estimate | SE | t.value | p-value |
| --- | --- | --- | --- | --- |
| (Intercept) | 0.233 | 157.9 | 0.01 | 0.99 |
| Age6 | 0.379 | 223.43 | 0.02 | 0.99 |
| Age9 | 1121.06 | 223.43 | 5.02 | 0.00015 |

F-statistic: 16.78 on 2 and 15 DF. R^2^=0.69

**Table S5.4** Summary statistics of the linear model (Gene~age) describing the relationship of the expression of the *hbb-i* gene from anemonefish (*Amphiprion melanopus*) larvae at ages 4, 6, and 9 days post-hatch (dph).

| Comparison (dph) | estimate | SE | t.value | p-value |
| --- | --- | --- | --- | --- |
| (Intercept) | 1.91 | 0.329 | 5.8 | <0.0001 |
| Age6 | 0.842 | 0.465 | 1.81 | <0.0904 |
| Age9 | -1.38 | 0.465 | -2.97 | 0.0095 |

F-statistic: 11.67 on 2 and 15 DF. R^2^=0.61

**Table S5.5** Summary statistics of the linear model (Gene~age) describing the relationship of the expression of the *hbb-ii* gene from anemonefish (*Amphiprion melanopus*) larvae at ages 4, 6, and 9 days post-hatch (dph).

| Comparison (dph) | estimate | SE | t.value | p-value |
| --- | --- | --- | --- | --- |
| (Intercept) | 0.158 | 0.0389 | 4.074 | 0.000997 |
| Age6 | 0.0976 | 0.055 | 1.78 | 0.0961 |
| Age9 | -0.103 | 0.055 | -1.88 | 0.0796 |

F-Statistic: 6.69 on 2 and 15 DF. R^2^=0.47

**Table S5.6** Summary statistics of the linear model (Gene~age) describing the relationship of the expression of the *hbb-iv* gene from anemonefish (*Amphiprion melanopus*) larvae at ages 4, 6, and 9 days post-hatch (dph).

| Comparison (dph) | estimate | SE | t.value | p-value |
| --- | --- | --- | --- | --- |
| (Intercept) | 1488.5 | 966.7 | 1.54 | 0.144 |
| Age6 | 2928.4 | 1367.2 | 2.14 | 0.049 |
| Age9 | 14803.1 | 1367.2 | 10.83 | <0.0001 |

F-statistic: 65.8 on 2 and 15 DF. R^2^=0.9

**Table S5.7** Summary statistics of the linear model (Gene~age) describing the relationship of the expression of the *ngb* gene from anemonefish (*Amphiprion melanopus*) larvae at ages 4, 6, and 9 days post-hatch (dph).

| Comparison (dph) | estimate | SE | t.value | p-value |
| --- | --- | --- | --- | --- |
| (Intercept) | 0.953 | 0.067 | 14.15 | <0.0001 |
| Age6 | 0.154 | 0.095 | 1.62 | 0.126 |
| Age9 | 0.438 | 0.095 | 4.6 | 0.00035 |

F-statistic: 10.89 on 2 an 15 DF. R^2^=0.59

**Table S5.8** Summary statistics of the linear model (Gene~age) describing the relationship of the expression of the *cytgb-ii* gene from anemonefish (*Amphiprion melanopus*) larvae at ages 4, 6, and 9 days post-hatch (dph).

| Comparison (dph) | Estimate | SE | t.value | p-value |
| --- | --- | --- | --- | --- |
| (Intercept) | 3.68 | 0.42 | 8.75 | <0.0001 |
| Age6 | -0.36 | 0.59 | -0.61 | 0.554 |
| Age9 | 1.91 | 0.59 | 3.23 | 0.0057 |

F-Statistic: 8.48 on 2 and 15 DF. R^2^=0.53

**Table S5.9** Summary statistics of the linear model (Gene~age) describing the relationship of the expression of the *mb* gene from anemonefish (*Amphiprion melanopus*) larvae at ages 4, 6, and 9 days post-hatch (dph).

| Comparison (dph) | Estimate | SE | t.value | p-value |
| --- | --- | --- | --- | --- |
| (Intercept) | 6.57 | 21.67 | 0.303 | 0.766 |
| Age6 | 111.39 | 30.65 | 3.63 | 0.0025 |
| Age9 | 202.4 | 30.65 | 6.6 | <0.0001 |

F-statistic: 21.88 on 2 and 15 DF. R^2^=0.74

**Table S5.10** Emmeans pairwise comparisons of the linear models (Gene~age) of the globins (*hba-i, hba-iii, hba-iv, hbb-I, hbb-ii, hbb-iv, ngb, cytgb, mb*) measured in our anemonefish (*Amphiprion melanopus*) larvae at 4, 6 and 9 days post hatch (dph)

| Gene | Comparison (dph) | estimate | SE | df | t.ratio | p-value |
| --- | --- | --- | --- | --- | --- | --- |
| *hba-i* | 4 – 6 | -17.43 | 13.6 | 15 | -1.28 | 0.43 |
| *hba-i* | 4 – 9 | -22.97 | 13.6 | 15 | -1.68 | 0.244 |
| *hba-i* | 6 – 9 | -5.52 | 13.6 | 15 | -0.404 | 0.914 |
| *hba-ii* | 4 – 6 | -19.6 | 9.28 | 15 | -2.11 | 0.122 |
| *hba-ii* | 4 – 9 | 14.8 | 9.28 | 15 | 1.59 | 0.279 |
| *hba-ii* | 6 – 9 | 34.3 | 9.28 | 15 | 3.7 | 0.0057 |
| *hba-iv* | 4 – 6 | -0.38 | 223 | 15 | -0.002 | 1.0 |
| *hba-iv* | 4 – 9 | -1121.06 | 223 | 15 | -5.02 | 0.0004 |
| *hba-iv* | 6 – 9 | -1120.68 | 223 | 15 | -5.02 | 0.0004 |
| *hbb-i* | 4 – 6 | -0.84 | 0.465 | 15 | -1.81 | 0.2 |
| *hbb-i* | 4 – 9 | 1.38 | 0.465 | 15 | 2.97 | 0.024 |
| *hbb-i* | 6 – 9 | 2.22 | 0.465 | 15 | 4.78 | 0.0007 |
| *hbb-ii* | 4 – 6 | -0.097 | 0.055 | 15 | -1.78 | 0.211 |
| *hbb-ii* | 4 – 9 | 0.103 | 0.055 | 15 | 1.88 | 0.179 |
| *hbb-ii* | 6 – 9 | 0.201 | 0.055 | 15 | 3.66 | 0.0062 |
| *hbb-iv* | 4 – 6 | -2928 | 1367 | 15 | -2.14 | 0.115 |
| *hbb-iv* | 4 – 9 | -14803 | 1367 | 15 | -10.83 | <0.0001 |
| *hbb-iv* | 6 – 9 | -11875 | 1367 | 15 | -8.69 | <0.0001 |
| *ngb* | 4 – 6 | -0.15 | 0.095 | 15 | -1.62 | 0.268 |
| *ngb* | 4 – 9 | -0.44 | 0.095 | 15 | -4.6 | 0.001 |
| *ngb* | 6 – 9 | -0.28 | 0.095 | 15 | -2.98 | 0.024 |
| *cytgb-ii* | 4 – 6 | 0.36 | 0.59 | 15 | 0.61 | 0.82 |
| *cytgb-ii* | 4 – 9 | -1.92 | 0.59 | 15 | -3.23 | 0.015 |
| *cytgb-ii* | 6 – 9 | -2.28 | 0.59 | 15 | -3.83 | 0.0044 |
| *mb* | 4 – 6 | -111 | 30.6 | 15 | -3.63 | 0.0065 |
| *mb* | 4 – 9 | -202 | 30.6 | 15 | -6.6 | <0.0001 |
| *mb* | 6 – 9 | -91 | 30.6 | 15 | -2.97 | 0.024 |

**Table S6.1** Model summary of the linear model (AAS~dph) describing the change in absolute aerobic scope (AAS; mg O_2_ kg^-1^ h^-1^) with age (days post hatch; dph) over the entire larval duration of the anemonefish (*Amphiprion melanopus*).

|  | Estimate | SE | t.value | p-value |
| --- | --- | --- | --- | --- |
| (Intercept) | 33.78 | 3.5 | 9.67 | <0.0001 |
| dph | -2.09 | 0.62 | -3.36 | 0.00124 |

F-statistic: 11.28 on 1 and 75 DF. R^2^=0.12

**Table S6.2** Emmeans pairwise comparisons of absolute aerobic scope (AAS; mg O_2_ kg^-1^ h^-1^) with age (days post-hatch; dph) over the entire larval duration of the anemonefish (*Amphiprion melanopus*)

| Comparison | Diff | Lwr | Upr | p-value |
| --- | --- | --- | --- | --- |
| 2dph – 1dph | 1467.1 | -20131.3 | 23065.5 | 0.999 |
| 3dph – 1dph | 1008.4 | -20590 | 22606.8 | 1.0 |
| 4dph – 1dph | 559.6 | -21664.9 | 22784.2 | 1.0 |
| 5dph – 1dph | -2387.7 | -23986.1 | 19210.7 | 0.999 |
| 6dph – 1dph | -19835.3 | -42059.8 | 2389.3 | 0.118 |
| 7dph – 1dph | -10664.8 | -32889.4 | 11559.7 | 0.834 |
| 8dph-1dph | -12536.8 | -33620.9 | 8547.2 | 0.613 |
| 9dph – 1dph | -9492.4 | -31717 | 12732.1 | 0.905 |
| 3dph – 2dph | -458.7 | -21412.2 | 20494.8 | 1.0 |
| 4dph – 2dph | -907.5 | -22505.9 | 20690.9 | 1.0 |
| 5dph – 2dph | -3854.8 | -24808.3 | 17098.8 | 0.999 |
| 6dph – 2dph | -21302.4 | -42900.8 | 296 | 0.0561 |
| 7dph – 2dph | -12131.9 | -33730.3 | 9466.5 | 0.682 |
| 8dph – 2dph | -14003.9 | -34426.9 | 6419 | 0.419 |
| 9dph – 2dph | -10959.5 | -32557.9 | 10638.9 | 0.787 |
| 4dph – 3dph | -448.8 | -22047.2 | 21149.6 | 1.0 |
| 5dph – 3dph | -3396 | -24349.6 | 17557.5 | 0.999 |
| 6dph – 3dph | -20843.6 | -42442 | 754.7 | 0.0668 |
| 7dph – 3dph | -11673.2 | -33271 | 9925.2 | 0.725 |
| 8dph – 3dph | -13545.2 | -33968.2 | 6877.8 | 0.466 |
| 9dph – 3dph | -10500.8 | -32099.2 | 11097.6 | 0.824 |
| 5dph – 4dph | -2947.3 | -24545.7 | 18651.1 | 0.999 |
| 6dph – 4dph | -20394.9 | -42619.4 | 1829.7 | 0.0972 |
| 7dph – 4dph | -11224.4 | -33448.9 | 11000.1 | 0.791 |
| 8dph – 4dph | -13096.4 | -34180.5 | 7987.6 | 0.555 |
| 9dph – 4dph | -10052 | -32276.6 | 12172.5 | 0.874 |
| 6dph – 5dph | -17447.6 | -39045.9 | 4150.8 | 0.211 |
| 7dph – 5dph | -8277.2 | -29875.6 | 13321.2 | 0.947 |
| 8dph – 5dph | -10149 | -30572.1 | 10273.8 | 0.806 |
| 9dph – 5dph | -7104.8 | -28703.2 | 14493.6 | 0.979 |
| 7dph – 6dph | 9170.4 | -13054.1 | 31395 | 0.921 |
| 8dph – 6dph | 7298.4 | -13785.6 | 28382.5 | 0.97 |
| 9dph – 6dph | 10342.8 | -11881.7 | 32567.4 | 0.856 |
| 8dph – 7dph | -1872 | -22956.1 | 19212.1 | 0.999 |
| 9dph – 7dph | 1172.4 | -21052.2 | 23396.9 | 1.0 |
| 9dph – 8dph | 3044.4 | -18039.7 | 24128.5 | 0.999 |

**Table S7.1** Model summary of the linear model (FAS~dph) describing the change in factorial aerobic scope (FAS) with age (days post hatch; dph) over the entire larval duration of the anemonefish (*Amphiprion melanopus*).

|  | Estimate | SE | t.value | p-value |
| --- | --- | --- | --- | --- |
| (Intercept) | 1.96 | 0.397 | 4.94 | <0.0001 |
| dph | 0.048 | 0.0705 | 0.684 | 0.50 |

F-Statistic: on 1 and 75 DF. R^2^=0.006

**Table S7.2** Emmeans pairwise comparisons of factorial aerobic scope (FAS) with age (days post-hatch; dph) over the entire larval duration of the anemonefish (*Amphiprion melanopus*)

| Comparison | Diff | Lwr | Upr | p-value |
| --- | --- | --- | --- | --- |
| 2dph – 1dph | 0.0817 | -2.43 | 2.59 | 1.0 |
| 3dph – 1dph | 1.29 | -1.22 | 3.8 | 0.775 |
| 4dph – 1dph | -0.0171 | -2.6 | 2.57 | 1.0 |
| 5dph – 1dph | 0.681 | -1.83 | 3.19 | 0.993 |
| 6dph – 1 dph | 0.00265 | -2.58 | 2.59 | 1.0 |
| 7dph – 1dph | 0.505 | -2.08 | 3.09 | 0.999 |
| 8dph – 1dph | 0.497 | -1.95 | 2.95 | 0.999 |
| 9dph – 1dph | 0.856 | -1.73 | 3.44 | 0.977 |
| 3dph – 2dph | 1.21 | -1.23 | 3.64 | 0.806 |
| 4dph – 2dph | -0.0987 | -2.61 | 2.41 | 1.0 |
| 5dph – 2dph | 0.6 | -1.84 | 3.04 | 0.997 |
| 6dph – 2dph | -0.079 | -2.59 | 2.43 | 1.0 |
| 7dph – 2dph | 0.423 | -2.09 | 2.93 | 0.999 |
| 8dph – 2dph | 0.415 | -1.96 | 2.79 | 0.999 |
| 9dph – 2dph | 0.774 | -1.74 | 3.29 | 0.985 |
| 4dph - 3dph | -1.31 | -3.82 | 1.2 | 0.762 |
| 5dph – 3dph | -0.61 | -3.05 | 1.83 | 0.996 |
| 6dph – 3dph | -1.29 | -3.8 | 1.22 | 0.777 |
| 7dph – 3dph | -0.787 | -3.3 | 1.72 | 0.984 |
| 8dph – 3dph | -0.794 | -3.17 | 1.58 | 0.976 |
| 9dph – 3dph | -0.435 | -2.95 | 2.08 | 0.999 |
| 5dph – 4dph | 0.698 | -1.81 | 3.21 | 0.992 |
| 6dph – 4dph | 0.0197 | -2.56 | 2.6 | 1.0 |
| 7dph – 4dph | 0.522 | -2.06 | 3.11 | 0.999 |
| 8dph – 4dph | 0.514 | -1.94 | 2.97 | 0.999 |
| 9dph – 4dph | 0.873 | -1.71 | 3.46 | 0.975 |
| 6dph – 5dph | -0.678 | -3.19 | 1.83 | 0.994 |
| 7dph – 5dph | -0.176 | -2.69 | 2.33 | 0.999 |
| 8dph – 5dph | -0.184 | -2.56 | 2.19 | 0.999 |
| 9dph – 5dph | 0.175 | -2.34 | 2.69 | 0.999 |
| 7dph – 6dph | 0.502 | -2.08 | 3.09 | 0.999 |
| 8dph – 6dph | 0.494 | -1.96 | 2.94 | 0.999 |
| 9dph – 6dph | 0.853 | -1.73 | 3.43 | 0.978 |
| 8dph – 7dph | -0.00773 | -2.46 | 2.44 | 1.0 |
| 9dph – 7dph | 0.351 | -2.23 | 2.93 | 0.999 |
| 9dph – 8dph | 0.359 | -2.09 | 2.81 | 0.999 |
