# Supplementary material for: Rapid physiological and transcriptomic changes associated with oxygen delivery in larval anemonefish suggest a role in adaptation to life on hypoxic coral reefs
Source: PLoS Biol. 2023 May 11;21(5):e3002102. doi: 10.1371/journal.pbio.3002102 (PMC10174562; doi:10.1371/journal.pbio.3002102)
Supplement: S1 Metadata — Description of metadata for each fish used in the study, and definition of variables. (DOCX) [file pbio.3002102.s004.docx]

**META-DATA** for Downie AT, Lefevre S, Illing B, Harris J, Jarrold M, McCormick MI, Nilsson GE, Rummer JL (submitted; PLoS Biology) Rapid physiological and transcriptomic changes associated with oxygen delivery in larval anemonefish suggest a role in adaptation to life on hypoxic coral reefs

**Methodology:** The study investigated how oxygen uptake rates, swimming performance, hypoxia tolerance and gene expression of proteins responsible for oxygen delivery and storage (e.g., hemoglobin, myoglobin, cytoglobin and neuroglobin) change as anemonefish larvae develop. Larvae were reared from adult anemonefishes, and daily measurements of swimming speed and oxygen uptake were taken (n=8-10 larvae per developmental day; 10 total developmental days for larval duration). An individual larva was placed in a swimming respirometer. A swimming respirometer is a glass chamber with an installed propellor that generates flow. The larvae swim against this flow, and oxygen probes measure the changes in oxygen uptake rates as the fish swim. The larvae would undergo a stepped velocity test (U_crit_; 1 body length per second every 30 minutes) until fatigue; this fatigue speed is the maximum swimming speed that the larvae can support mainly aerobically. At each velocity increment until fatigue, oxygen uptake rate is measured. The oxygen uptake rate at maximum swimming speed is Maximum Metabolic Rate (MMR). Extrapolating the oxygen uptake rates at each swimming speed to '0' swimming speed provides an estimate of Standard Metabolic Rate (SMR), which are basic maintenance costs. The difference between MMR and SMR is aerobic scope (AS), which is an estimate for whole-animal energy budgets. We found that there was a distinct decrease in oxygen uptake rates from 4 days post-hatch (dph) to 6dph, which remained constant until settlement (9dph). We measured hypoxia tolerance by placing larvae at ages 4, 6 and 9dph (n=8-14) individually into small jars and measuring the point where larvae lose equilibrium due to loss of oxygen in the jar by the larvae’s respiration. Additionally, six larvae were selected at each of these three developmental days (4,6 and 9dph), and using established techniques, their RNA was extracted. The mRNA was sequenced to investigate changes in genes coding for proteins associated with oxygen delivery and transport (hemoglobin and myoglobin), and oxygen storage (e.g., neurglobin and cytoglobin).

The supplementary data material consists of 4 files in comma-separated values (.csv) format (used for statistical analyses), 1 file as an Excel workbook (with 12 workbook pages), and a metadata file (.doc) that includes all of the variables, labels and units.

The data files are:

**Downie et al_PLOS Biology_MetaData.doc**

This File

**Supplementary Data File 1_Gene Expression.xlsx**

Expression data and statistical results from transcriptomic analyses of anemonefish larvae sampled at 4, 6 and 9 days post hatch

**Supplementary Data File 2_Ucrit_RAW.csv**

Daily measurements of swimming speed of larval anemonefish

**Supplementary Data File 3_MO2_RAW.csv**

Daily measurements of standard metabolic rate, maximum metabolic rate, absolute aerobic scope, and factorial aerobic scope of larval anemonefish

**Supplementary Data File 4_hypoxia_RAW.csv**

Measure of hypoxia tolerance of larval anemonefish at 4, 6 and 9 days post hatch

**Supplementary Data File 5_Gene_RAW.csv**

Measure of gene expression of hemoglobin, myoglobin, neuroglobin and cytoglobin for larval anemonefishes at 4, 6 and 9dph.

.csv headings and units

**ID**

Unique identification of each individual fish; ID composed of date sampled and individual number for that specific day (MO2) or age and individual number (gene expression)

**Fish#**

Number associated with that individual per day; same as number associated with ID

**Date**

Date (month.day_year) that experiment was performed

**dph**

age of the fish (days post hatch); dph of 0 indicates day of hatching

**age**

age of fish (days post hatch)

**Mass Range**

Categorization of individual fish under a mass range (in milligrams)

**Mass**

Mass of individual larva in grams

**Mass** (mg)

Mass of individual larva in milligrams

**Mass (kg)**

Mass of individual in kilograms (convert for MO2)

**Length (cm)**

Total length of individual in cm

**Length Range**

Categorization of individual fish under a length range (in cm)

**U_crit_**

Critical swimming speed of the fish after swimming (cm/s)

**Ucrit_BL**

Critical swimming speed of the fish after swimming (BL/s)

**raw.SMR**

standard metabolic rate not adjusted for mass (mg O_2_ h^-1^)

**raw.MMR**

maximum metabolic rate not adjusted for mass (mg O_2_ h^-1^)

**mass.SMR**

Standard metabolic rate adjusted for mass (mg O_2_ kg^-1^ h^-1^)

**Mass.SMR.g**

Standard metabolic rate adjusted for mass (mg O_2_ g^-1^h^-1^)

**mass.MMR**

Maximum metabolic rate adjusted for mass (mg O_2_ kg^-1^ h^-1^)

Mass.MMR.g

Maximum metabolic rate adjusted for mass (mg O_2_ g^-1^h^-1^)

**AAS**

Absolute aerobic scope (mg O_2_ kg^-1^ h^-1^; mass.MMR – mass.SMR)

**AAS.g**

Absolute aerobic scope (mg O2 g^-1^h^-1^; mass.MMR.g-mass.SMR.g)

**FAS**

Factorial aerobic scope (mass.MMR/mass.SMR)

**LOE**

Loss of equilibrium; percentage (%) of oxygen in water that results in larvae to flip over, indicating hypoxia tolerance

**DO2**

Amount of dissolved oxygen in water (mg O_2_ ml^-1^) at 28°C at LOE

***hba-i***

hemoglobin subunit alpha paralog i gene expression

***hba-ii***

hemoglobin subunit alpha paralog ii gene expression

***hba-iv***

hemoglobin subunit alpha paralog iv gene expression

***hbb-i***

hemoglobin subunit beta paralog I gene expression

***hbb-ii***

hemoglobin subunit beta paralog ii gene expression

***hbb-iv***

hemoglobin subunit beta paralog iv gene expression

***mb***

myoglobin gene expression

***ngb***

neuroglobin gene expression

***cygb-ii***

cytoglobin paralog ii gene expression

.xlsx workbook page summary

Page 1. all_degs_getmm

GeTMM-normalised expression and gene IDs (with abbreviations and descriptions) for genes identified as differentially expressed (DEGs), for each individual fish larvae across three age groups (4, 6 and 9dph) (Figure 3B)

Page 2. Heatmap_toptags

GeTMM-normalised expression and gene IDs (with abbreviations and descriptions) for the top most significant genes across three age groups (4, 6 and 9dph), used to create Heatmap (Figure 3D)

Page 3. Volcanoplot_toptag

Mean across all groups (‘baseMean’), log10 of the mean (‘log10basemean_amel’), log of the fold-change (‘log2FoldChange’), error estimate of the log fold-change (lfcSE), significance (‘pvalue’) and adjusted p value (‘padj’) for the top most significant DEGs when comparing 9 days to 4 days post hatch, used to create Volcano plot (Figure 3E)

Page 4a. 6dph_vs_4dph, 4b. 9dph_vs_4dph, 4c. 9dph_vs_6dph

Mean across all groups (‘baseMean’), log10 of the mean (‘log10basemean_amel’), log of the fold-change (‘log2FoldChange’), error estimate of the log fold-change (lfcSE), significance (‘pvalue’) and adjusted p value (‘padj’) for all DEGs identified when comparing 9dph and 4dph, 9dph and 6 dph, and 6dph and 4dph.

Page 5a. down_unique_6dph_vs_4dph, Page 5b. up_unique_6dph_vs_4dph

Mean across all groups (‘baseMean’), log10 of the mean (‘log10basemean_amel’), log of the fold-change (‘log2FoldChange’), error estimate of the log fold-change (lfcSE), significance (‘pvalue’) and adjusted p value (‘padj’) for DEGs that were unique when comparing 6dph and 4dph , i.e. down or up at 6dph but not 9 dph

Page 6a. down_unique_9dph_vs_6dph, Page 6b. up_unique_9dph_vs_6dph

Mean across all groups (‘baseMean’), log10 of the mean (‘log10basemean_amel’), log of the fold-change (‘log2FoldChange’), error estimate of the log fold-change (lfcSE), significance (‘pvalue’) and adjusted p value (‘padj’) for DEGs that were unique when comparing 9dph and 6dph, i.e. down or up at 9dph but not at 6dph

Page 7. GOs

Gene ontology enrichment analyses (of the significantly up- or down-regulated genes when comparing 9dph and 4dph. Data includes the fold enrichment (how many more times than expected did a gene belonging to a given GO category appear in the list of DEGs) and the adjusted p-value (FDR).

Page 8. Haemoglobin_getmm

GeTMM-normalised expression and gene IDs for hemoglobin (alpha and beta subunits and paralogs i, ii, and iv), myoglobin, cytoglobin and neuroglobin for each individual larval anemonefish at ages 4, 6 and 9 dph. This data is the same as ‘Supplementary Data File 5_GENE RAW.csv’.

Page 9. Raw_counts

Raw, non-normalised gene counts for all genes from the FeatureCount pipeline
