# Supplementary figures and images for: Rapid physiological and transcriptomic changes associated with oxygen delivery in larval anemonefish suggest a role in adaptation to life on hypoxic coral reefs
Source: PLoS Biol. 2023 May 11;21(5):e3002102. doi: 10.1371/journal.pbio.3002102 (PMC10174562; doi:10.1371/journal.pbio.3002102)

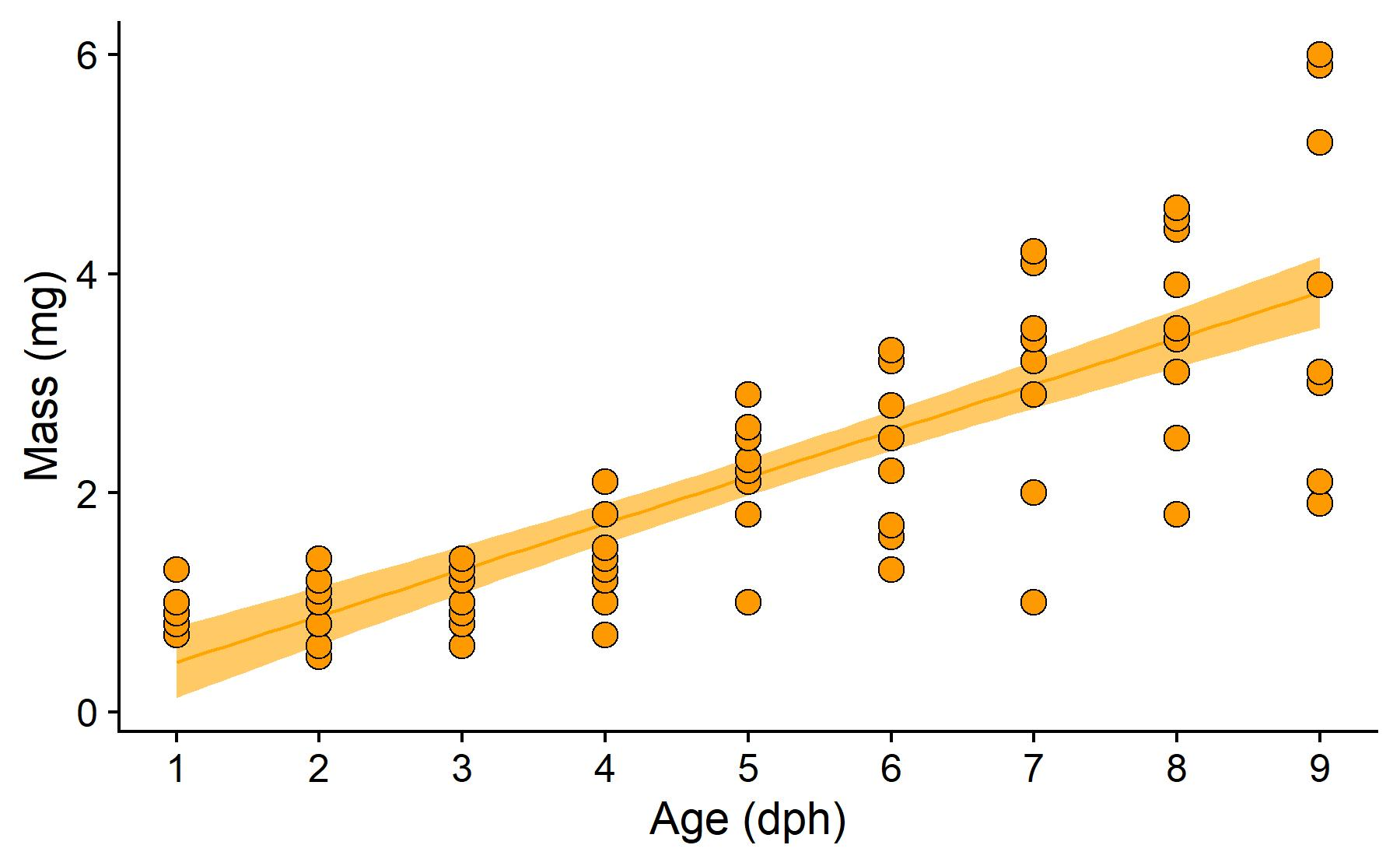

Supplement: S1 Fig — Relationship between age (dph) and mass (mg) for the anemonefish (Amphiprion melanopus) over the entire larval duration (1–9 dph; n = 8–10 individuals per day) during swimming respirometry experiments. LMs are present with 95% confidence intervals. The regression equation is based on the best fitting LM: mass = 0.422(age)+0.0328; r2 = 0.65). The data underlying this figure can be found on sheet 2 in S1 Data. dph, days post hatch; LM, linear model. (TIF) [file pbio.3002102.s005.tif]

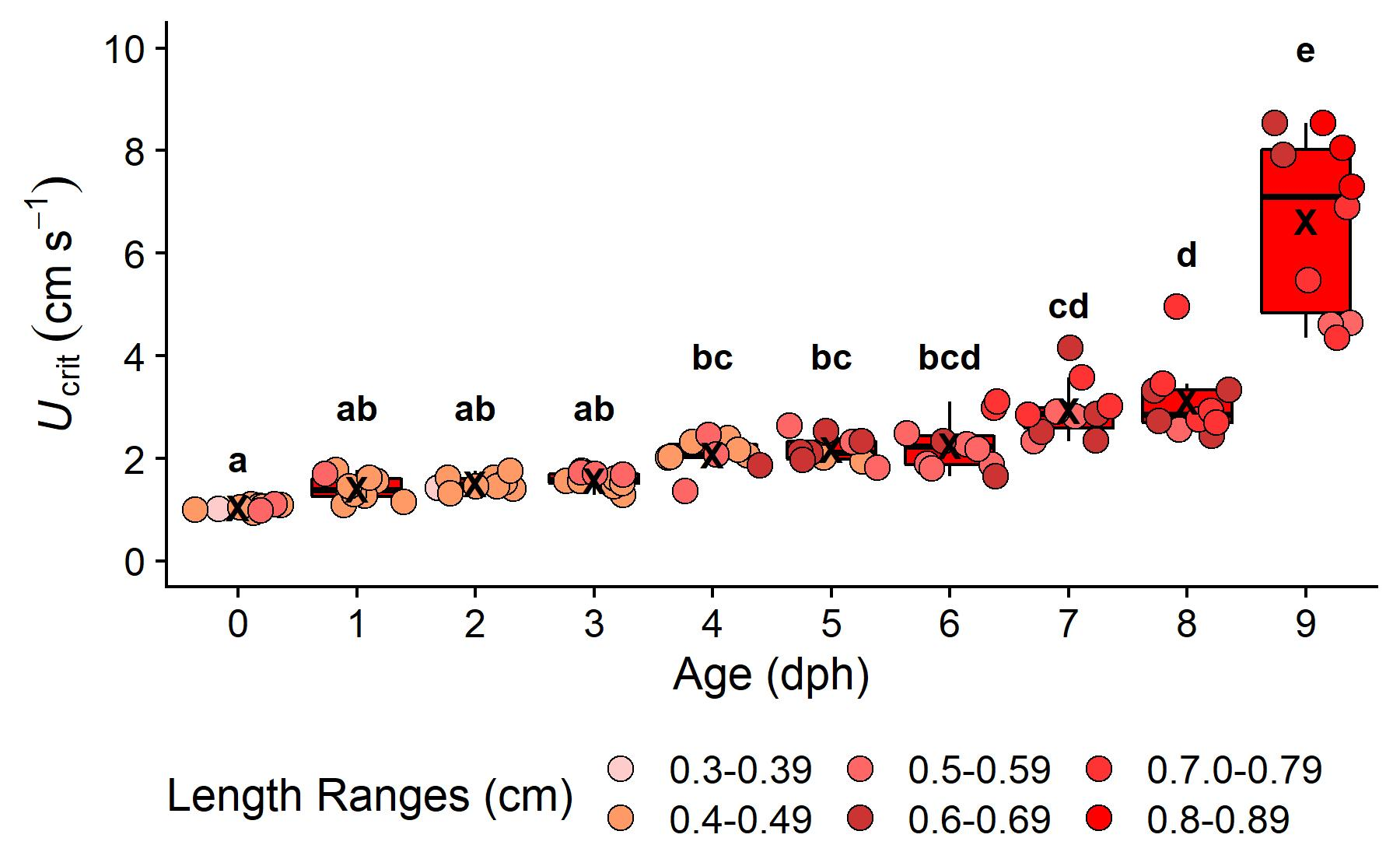

Supplement: S2 Fig — Relationship between age (dph) and critical swimming speed (cm s−1) for the anemonefish (Amphiprion melanopus) over the entire larval duration (1–9 dph; n = 8–10 individuals per day) during swimming respirometry experiments). Each point is colour coded to represent the size range (length; cm) of the individual larva. Boxplots show median and interquartile ranges, and “X” indicates average age (dph), and different lowercase letters represent statistical differences (LMs; α = 0.05). The data underlying this figure can be found on sheet 1 in S1 Data, and details on statistical output can be found in the supporting information S1 StatisticalOutput. dph, days post hatch; LM, linear model. (TIF) [file pbio.3002102.s006.tif]

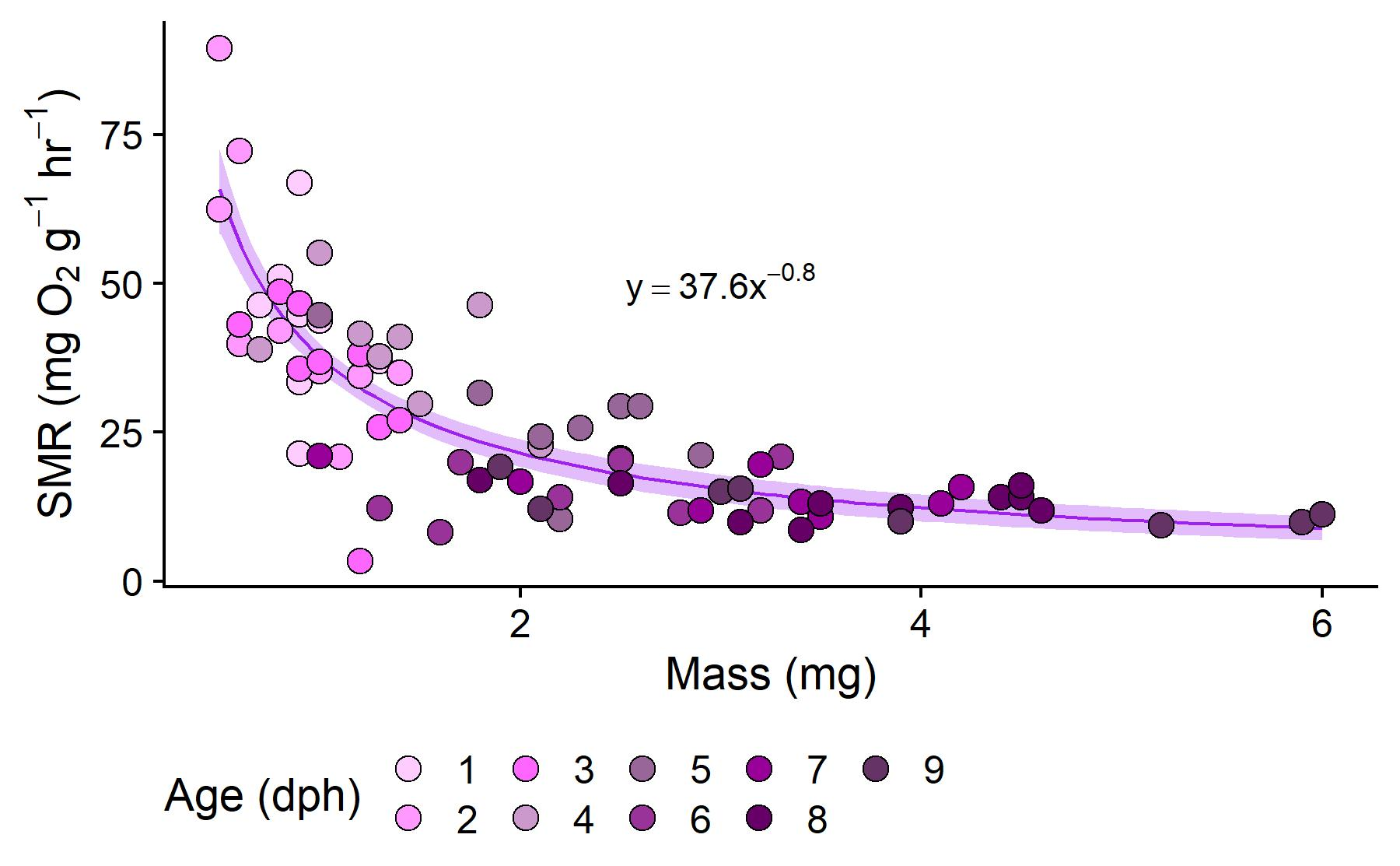

Supplement: S3 Fig — Relationship between mass-specific SMR (mg O2 g−1 h−1) and mass (mg) for the anemonefish (Amphiprion melanopus) over the entire larval duration (1–9 dph; n = 8–10 individuals per day) during swimming respirometry experiments (see Methods for details). Each point is colour coded to represent the age (dph) of the individual larva. Model predictions are presented with 95% confidence intervals. The equation for the exponential curve fit is present on the figure showing the scaling exponent for how mass-specific SMR is predicted to change with mass (r2 = 0.47). The data underlying this figure can be found on sheet 2 in S1 Data. dph, days post hatch; SMR, standard metabolic rate. (TIF) [file pbio.3002102.s007.tif]

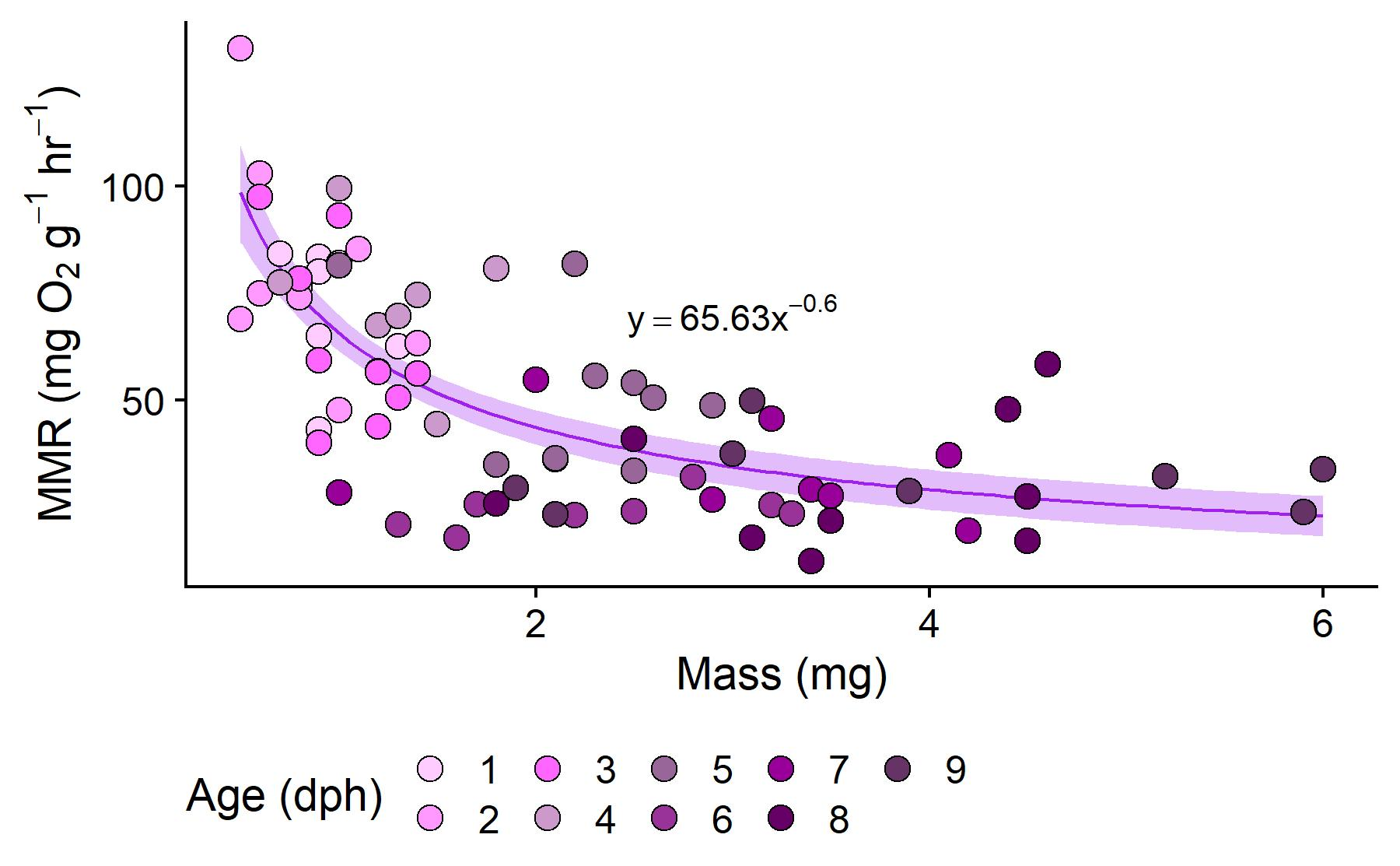

Supplement: S4 Fig — Relationship between mass-specific MMR (mg O2 g−1 h−1) and mass (mg) for the anemonefish (Amphiprion melanopus) over the entire larval duration (1–9 dph; n = 8–10 individuals per day) during swimming respirometry experiments (see Methods for details). Each point is colour coded to represent the age (dph) of the individual larva. Model predictions are presented with 95% confidence intervals. The equation for the exponential curve fit is present on the figure showing the scaling exponent for how mass-specific MMR is predicted to change with mass (r2 = 0.39). The data underlying this figure can be found on sheet 2 in S1 Data. dph, days post hatch; MMR, maximum metabolic rate. (TIF) [file pbio.3002102.s008.tif]

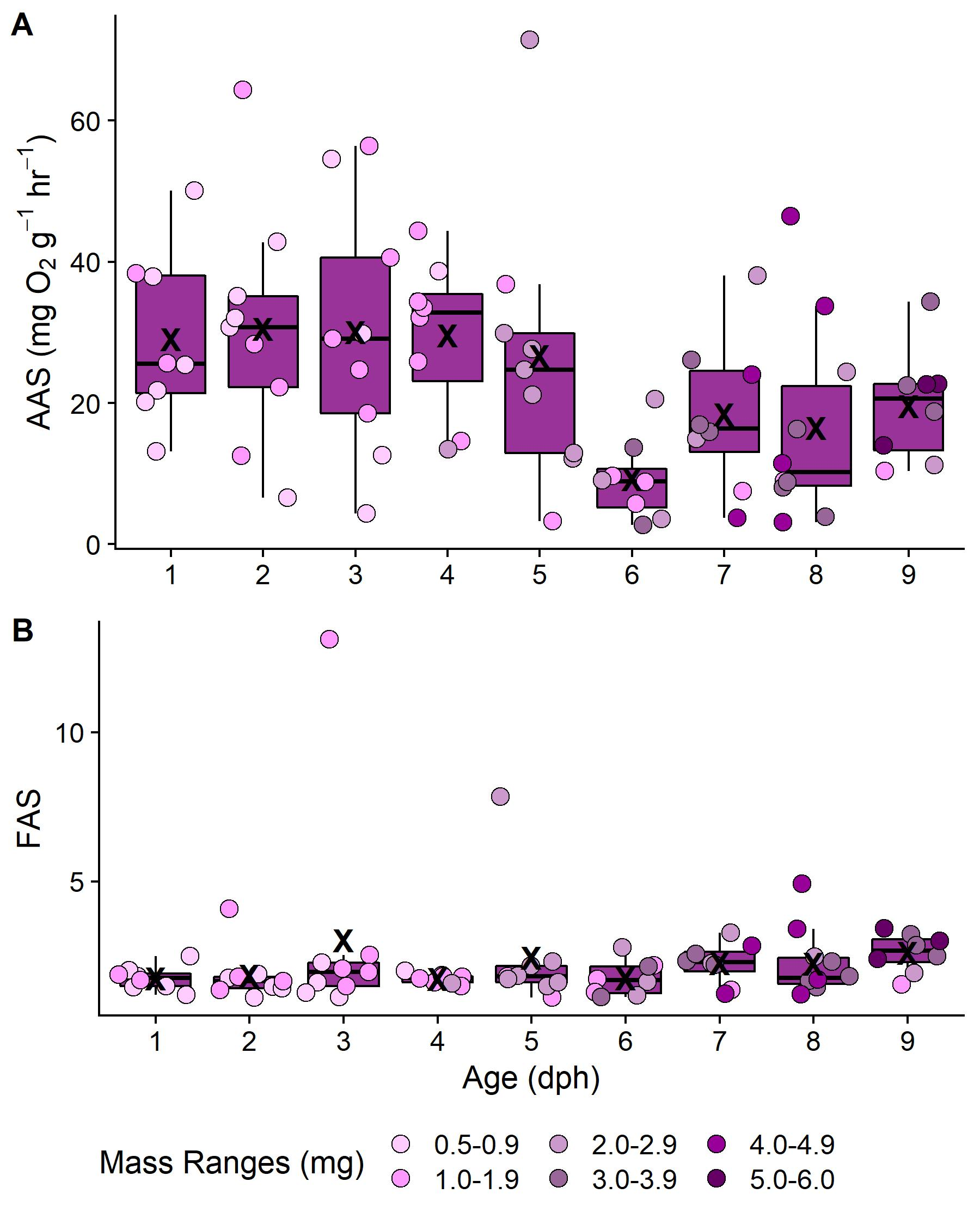

Supplement: S5 Fig — Relationship between (A) AAS (mg O2 g−1 h−1) and (B) FAS with age (dph) over the entire larval duration of the anemonefish (Amphiprion melanopus). Each individual point represents calculations from oxygen uptake rates from each individual larva (n = 8–10 per age) swum under a swimming respirometry protocol (see Methods for details) to achieve simultaneous measures of swimming speed and oxygen uptake rates. Each point is colour coded to represent the size range (mass; mg) of the individual larva. Boxplots show median and interquartile ranges, and “X” indicates average AAS or FAS per age (dph). The data underlying this figure can be found on sheet 2 in S1 Data, and details on statistical output can be found in the supporting information S1 StatisticalOutput. AAS, absolute aerobic scope; dph, days post hatch; FAS, factorial aerobic scope. (TIF) [file pbio.3002102.s009.tif]
